# Pathologic response after total neoadjuvant therapy in stage II–III rectal cancer: preliminary results from a prospective study from Vietnam

**DOI:** 10.3389/fonc.2026.1774856

**Published:** 2026-03-24

**Authors:** Vo Duc Hieu, Phan Thi Hong Duc, Nguyen Hoang Quy, Vo Ngoc Huan, Le Quoc Khanh, Le Hoang Dinh Nguyen, Pham Thi Minh Thu, Pham Hung Cuong

**Affiliations:** 1 Ho Chi Minh City Oncology Hospital, Ho Chi Minh City, Vietnam; 2 Department of Oncology, Pham Ngoc Thach University of Medicine, Ho Chi Minh City, Vietnam; 3 Department of Oncology, University of Medicine and Pharmacy at Ho Chi Minh City, Ho Chi Minh City, Vietnam

**Keywords:** CapeOX, chemoradiotherapy, FOLFOX, pathological complete response, rectal cancer, total neoadjuvant therapy, tumor regression

## Abstract

**Introduction:**

Total neoadjuvant therapy (TNT) is increasingly recommended for locally advanced rectal cancer (LARC), yet data from developing countries remain limited. In Vietnam, constraints in diagnostic access, radiotherapy availability, and multidisciplinary coordination may affect treatment feasibility and outcomes.

**Objective:**

To evaluate pathological response, safety, and perioperative outcomes of TNT for stage II–III rectal cancer in a Vietnamese oncology center.

**Methods:**

This prospective single-arm study enrolled 101 patients who received long-course chemoradiotherapy followed by consolidation capecitabine–oxaliplatin (CAPOX) or 5-fluorouracil/leucovorin–oxaliplatin (FOLFOX)before total mesorectal excision. Pathologic response and tumor regression grade were assessed.

**Results:**

Among 95 resected patients, pathological complete response (pCR) was 35.7%, and good regression (TRG 0–1) was achieved in 70.4%. Treatment completion was high (94.1%), with acceptable toxicity and no treatment-related mortality.

**Conclusion:**

TNT is feasible, safe, and achieves response outcomes comparable to global benchmarks, supporting its integration into rectal cancer management in resource-limited settings.

## Introduction

Colorectal cancer ranks third in global incidence and fourth in cancer-related mortality, representing a major public health challenge worldwide (1). Within this group, locally advanced rectal cancer (LARC) is defined as stage II disease (cT3–4, N0) or stage III disease (node-positive) (2). The therapeutic goals for LARC are not only to achieve locoregional control but also to prevent distant metastases, while preserving function and improving patients’ quality of life.

The degree of tumor response following neoadjuvant therapy is a key prognostic indicator in rectal cancer. Pathologic response was assessed according to the modified Ryan Tumor Regression Grade (TRG) postoperatively, treatment outcomes can be classified as complete response (CR) — defined by the absence of viable tumor cells pathologically (TRG 0); partial response (PR) — indicating substantial but incomplete tumor regression with residual viable cells (TRG 1–2) (3–7).

Among these, achieving a pathological complete response (pCR) is strongly correlated with improved disease-free survival and overall survival, and may enable organ-preserving strategies such as “watch-and-wait” in selected patients (8–10). Therefore, enhancing pCR rates through optimized treatment sequencing and systemic intensification has become a major focus in modern rectal cancer management (8–10).

For decades, the standard treatment paradigm has consisted of long-course chemoradiotherapy (LC-CRT) followed by total mesorectal excision (TME) and postoperative adjuvant chemotherapy (11). However, compliance with and completion of adjuvant chemotherapy after surgery is often suboptimal because of treatment-related toxicity and postoperative complications, which limits its efficacy in controlling distant disease. Moreover, conventional long-course chemoradiotherapy yields a modest pathological complete response (pCR) rate of 10–30%, with local recurrence of 5–10% and distant metastasis of 25–30% within 5 years (2, 11). To address these limitations, total neoadjuvant therapy (TNT)—the delivery of the entire course of systemic chemotherapy in addition to chemoradiotherapy before surgery—has been proposed and increasingly adopted. Meta-analyses of randomized controlled trials have shown that TNT significantly increases the pCR rate (odds ratio [OR] 1.77; 95% confidence interval [CI], 1.28–2.45; p = 0.0005), improves disease-free survival (DFS) (hazard ratio [HR] 0.83; 95% CI, 0.72–0.96), and reduces the risk of distant metastasis (HR 0.81; 95% CI, 0.68–0.95) compared with standard LC-CRT (12).

In TNT protocols, radiotherapy remains a central component — either as long-course chemoradiotherapy (LC-CRT) or short-course radiotherapy (SC-RT) — followed by consolidation or induction systemic chemotherapy. The incorporation of radiotherapy prior to surgery is intended to maximize local tumor control and enhance chemosensitivity via radiation-induced vascular remodeling and immunogenic cell death, thereby increasing the likelihood of achieving a pathologic complete response (pCR) (13–16). Several trials comparing TNT regimens with traditional postoperative adjuvant chemotherapy have addressed the question of surgical safety: pooled analyses demonstrate no significant increase in perioperative morbidity or mortality with TNT, and some studies even report lower rates of postoperative complications and higher completion rates of systemic therapy than in the adjuvant setting (17, 18). These findings support the feasibility and safety of “surgery after TNT” as an effective alternative to the older paradigm of “surgery then adjuvant chemotherapy”.

Although total neoadjuvant therapy (TNT) is now endorsed by major international guidelines for locally advanced rectal cancer, its real-world implementation in resource-limited settings remains uncertain. In Vietnam—where access to modern radiotherapy techniques, perioperative multidisciplinary care, and chemotherapy delivery can be constrained—there are scant prospective data on feasibility, pathologic response, and safety. In the current study, we set out to investigate the pathologic response and perioperative morbidity of a Total Neoadjuvant Therapy strategy—specifically long-course chemoradiotherapy followed by preoperative chemotherapy—in patients with stage II–III rectal cancer treated at a specialized cancer hospital in Vietnam, eventually comparing the outcomes against benchmarks from developed countries and high-resource settings.

## Materials and methods

### Study design

This was an observational, prospective, single-arm, single-center study conducted at Ho Chi Minh City Oncology Hospital, Vietnam.

### Patient selection

All patients aged 18 years or older with stage II–III rectal adenocarcinoma admitted to the Department of General Radiotherapy, Ho Chi Minh City Oncology Hospital, between June 2023 and April 2024 were eligible for inclusion. Patients were required to have a preoperative diagnosis of stage II–III rectal cancer with histologically confirmed adenocarcinoma, an indication for neoadjuvant chemoradiotherapy, ECOG performance status 0–1 and written informed consent. Patients with rectal cancer complicated by obstruction or perforation, multiple synchronous rectal tumors, or concomitant malignancies were excluded.

### Treatment

The investigational treatment consisted of standard long-course chemoradiotherapy with a total dose of 50 Gy in 25 fractions combined with concurrent capecitabine as a radiosensitizer, followed by 6 cycles of capecitabine–oxaliplatin (CAPOX) or 9 cycles of 5 fluorouracil/leucovorin–oxaliplatin (FOLFOX). At the end of neoadjuvant treatment, patients underwent restaging with Doppler ultrasound of the neck, pelvic MRI, and colonoscopy. Radiologic response was assessed by MRI according to RECIST version 1.1, and classified as complete response, partial response, stable disease, or progressive disease Patients without evidence of distant metastasis or disease progression subsequently underwent radical surgery with total mesorectal excision. Pathologic complete response (pCR): defined as the absence of viable tumor cells in both primary tumor and regional lymph nodes in the surgical specimen, confirmed on hematoxylin–eosin (H&E)–stained sections by light microscopic examination.

### Follow-up

Postoperative follow-up was performed including history and physical examination and serum CEA every 3 months for the first 2 years and every 6 months until year 5. MRI pelvis was obtained every 12 months for 5 years in stage II–III patients. Colonoscopy was scheduled 1 year after surgery, then repeated at 3 years and every 5 years thereafter.

### Statistics

Data from case report forms (CRFs) were entered into a dedicated data management system and verified against source documents prior to analysis. Continuous variables were summarized using mean ± standard deviation or median (interquartile range), as appropriate, and categorical variables were presented as frequencies and percentages. Comparisons between categorical variables were performed using the chi-square test or Fisher’s exact test, as appropriate. Associations between baseline factors and pathologic complete response (pCR) were assessed using Fisher’s exact test for categorical variables. Effect sizes were reported as crude odds ratios (ORs) with exact 95% confidence intervals (CIs). Variables with p < 0.25 in univariate analysis were considered for inclusion in multivariate logistic regression. Time-to-event outcomes, including recurrence-free survival (RFS), were estimated using the Kaplan–Meier method, with patients censored at the time of last follow-up if no event had occurred. All statistical tests were two-sided, and p < 0.05 was considered statistically significant. Analyses were performed using SPSS software version 20.0 (IBM Corp., Armonk, NY, USA).

## Results

### Study population

Between June 2023 and April 2024, a total of 101 patients with stage II–III rectal adenocarcinoma were enrolled. Baseline patient and tumor characteristics are summarized in (**Table 1**). The cohort had a predominantly good performance status. Most tumors were located in the mid rectum and were moderately differentiated. The majority of patients presented with locally advanced disease, with more than half having cT4 tumors and most demonstrating nodal involvement, frequently at an advanced nodal stage. A substantial proportion also showed high-risk features, including mesorectal fascia involvement and extramural vascular invasion. In 34 patients achieving pCR, LVI/PNI were not applicable due to absence of viable tumor.

**Table 1:** Baseline characteristics of the study population (N = 101).

Characteristic	Value
Median age, years (range)	55 (22–74)
Sex, n (%)	Male: 61(60.4%) Female: 40 (39.6%)
ECOG performance status, n (%)	0: 92(91.1%) 1: 9 (8.9%)
Tumor distance from anal verge, mm (range)	58 (0–170)
Tumor location by distance from anal verge, n (%)	Lower rectum (<5 cm): 34 (33.7%) Mid rectum (5–10 cm): 49 (48.5%) Upper rectum (>10 cm): 18 (17.8%)
Histologic grade, n (%)	Grade 1: 5 (5%) Grade 2: 93 (92%) Grade 3: 3 (3%)
Clinical T stage, n (%)	T3: 47 (46.5%) T4: 54 (53.5%)
Clinical N stage, n (%)	N0: 9 (8.9%) N1: 28 (27.7%) N2a: 43 (42.6%) N2b: 21 (20.8%)
AJCC stage, n (%)	IIA: 5 (5.0%) IIB: 3 (3.0%) IIC: 1 (1.0%) IIIA: 0 (0%) IIIB: 49 (48.5%) IIIC: 43 (42.5%)
Mesorectal fascia involvement (MRF), n (%)	≤1 mm: 36 (35.6%) >1 mm: 44 (43.6%) Not assessed: 21 (20.8%)
Extramural vascular invasion (EMVI), n (%)	Positive: 29 (28.7%) Negative: 51 (50.5%) Not assessed: 21 (20.8%)

### Treatment and surgical outcomes

Treatment delivery and surgical outcomes are summarized in (**Table 2**). All patients received long-course chemoradiotherapy with 50 Gy in 25 fractions and concurrent capecitabine, followed by consolidation chemotherapy. CAPOX was the predominant consolidation regimen (99%); two patients did not complete all six cycles of CAPOX because of toxicity. Most patients proceeded to surgery, with a high rate of sphincter preservation (72.3%). Six patients did not proceed to surgery—two declined resection, while four developed distant metastases during TNT. Among resected patients, an R0 resection was achieved in 97.9%, indicating excellent oncologic clearance.

**Table 2:** Effectiveness of TNT and surgical outcomes.

Characteristics	n (%)
Disease progression during treatment (n=101)	
Neoadjuvant concurrent chemoradiotherapy	0 (0%)
Chemotherapy	4 (4%)
Surgery method (n=97)	
Sphincter-preserving surgery	73 (75.3%)
Non-sphincter-preserving surgery	22 (22.7%)
Refused surgery	2 (2%)
Resection margin (n=95)	
R0	93 (97.9%)
R1	0 (0%)
R2	2 (2.1%)
ypT (n=95)	
ypT0	44 (46.3%)
ypT1	5 (5.3%)
ypT2	20 (21.1%)
ypT3	23 (24.2%)
ypT4	3 (3.1%)
ypN (n=95)	
ypN0	83 (87.4%)
ypN1	9 (9.5%)
ypN2	3 (3.1%)
ypM (n=95)	
M0	93 (98%)
M1	2 (2%)
ypT0N0 (n=95)	
Yes	34 (35.7%)
Tumor regression (AJCC) (n=95)	
TRG 0	34 (35.7%)
TRG 1	33 (34.7%)
TRG 2	19 (20%)
TRG 3	9 (9.6%)
ypLVI (n=61)	
Positive	6 (9.8%)
Negative	54 (88.6%)
Not determined	1 (1.6%)
ypPNI (n=61)	
Positive	7 (11.5%)
Negative	53 (86.9%)
Not determined	1 (1.6%)

### Pathologic response

**Figure 1:**
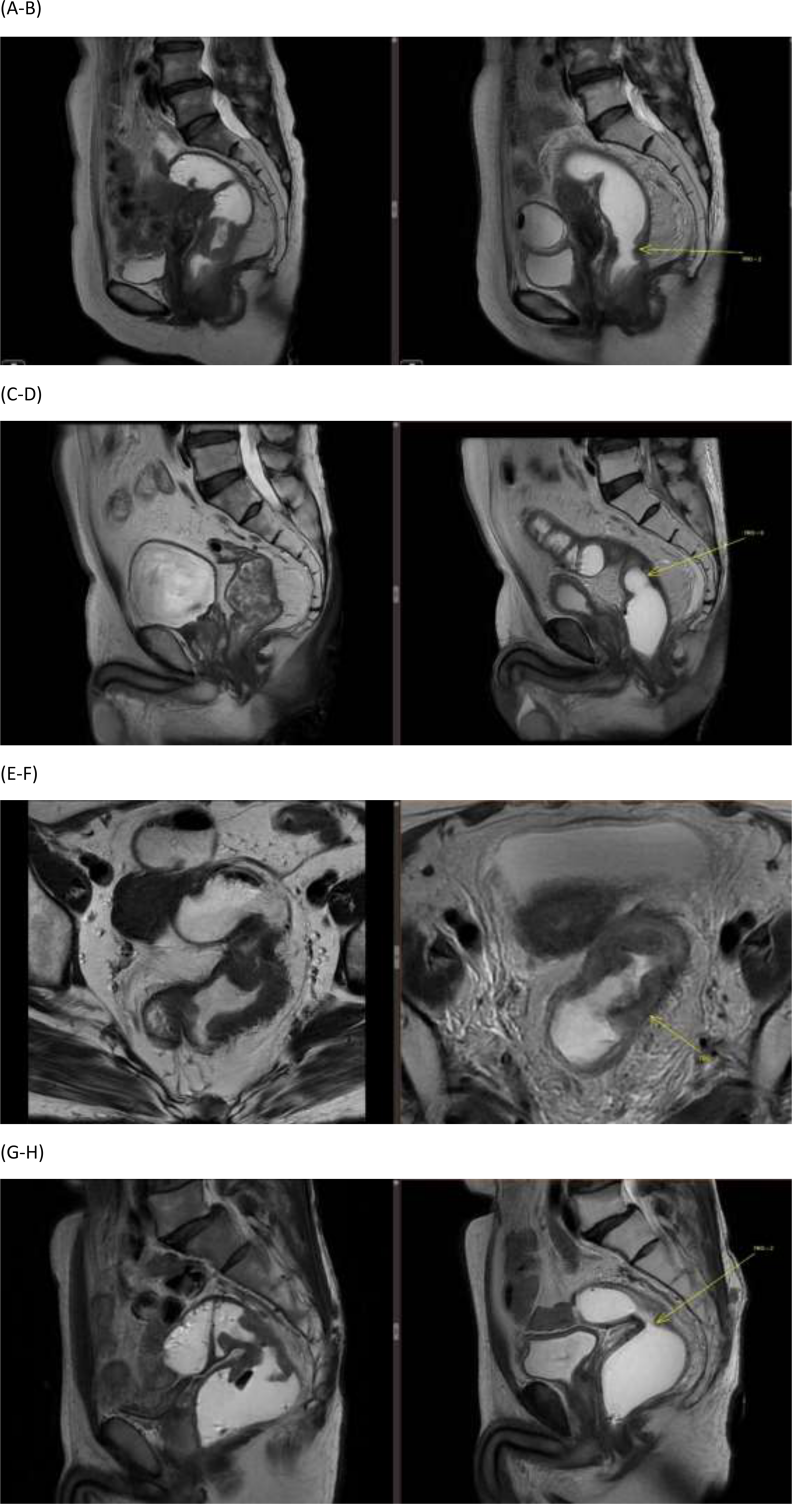
MRI assessment of tumor response before total neoadjuvant therapy (TNT) and at preoperative restaging after TNT (prior to surgery). **(A, B)** Sagittal T2-weighted MRI before TNT and at preoperative restaging after TNT showing partial response, confirmed by pathologic TRG = 2. **(C, D)** Sagittal T2-weighted MRI before TNT and at preoperative restaging after TNT showing complete response, confirmed by pathologic TRG = 0 **(E, F)** Axial T2-weighted MRI before TNT and at preoperative restaging after TNT showing partial response (PR), confirmed by pathologic TRG = 2. **(G, H)** Sagittal T2-weighted MRI before TNT and at preoperative restaging after TNT showing partial response, confirmed by pathologic TRG = 2.

Pathologic outcomes demonstrated substantial tumor downstaging and regression following TNT (**Table 2**, **Figure 1**). Nearly half of the resected patients achieved complete eradication of the primary tumor (ypT0), and nodal sterilization was observed in the majority, with 87.4% classified as ypN0. Overall, pathological complete response (ypT0N0) was achieved in 35.7% of patients. Tumor regression grading further supported these findings, with 70.4% demonstrating good pathological response (TRG 0–1), indicating a high rate of meaningful tumor regression after neoadjuvant intensification.

**Figure 2:**
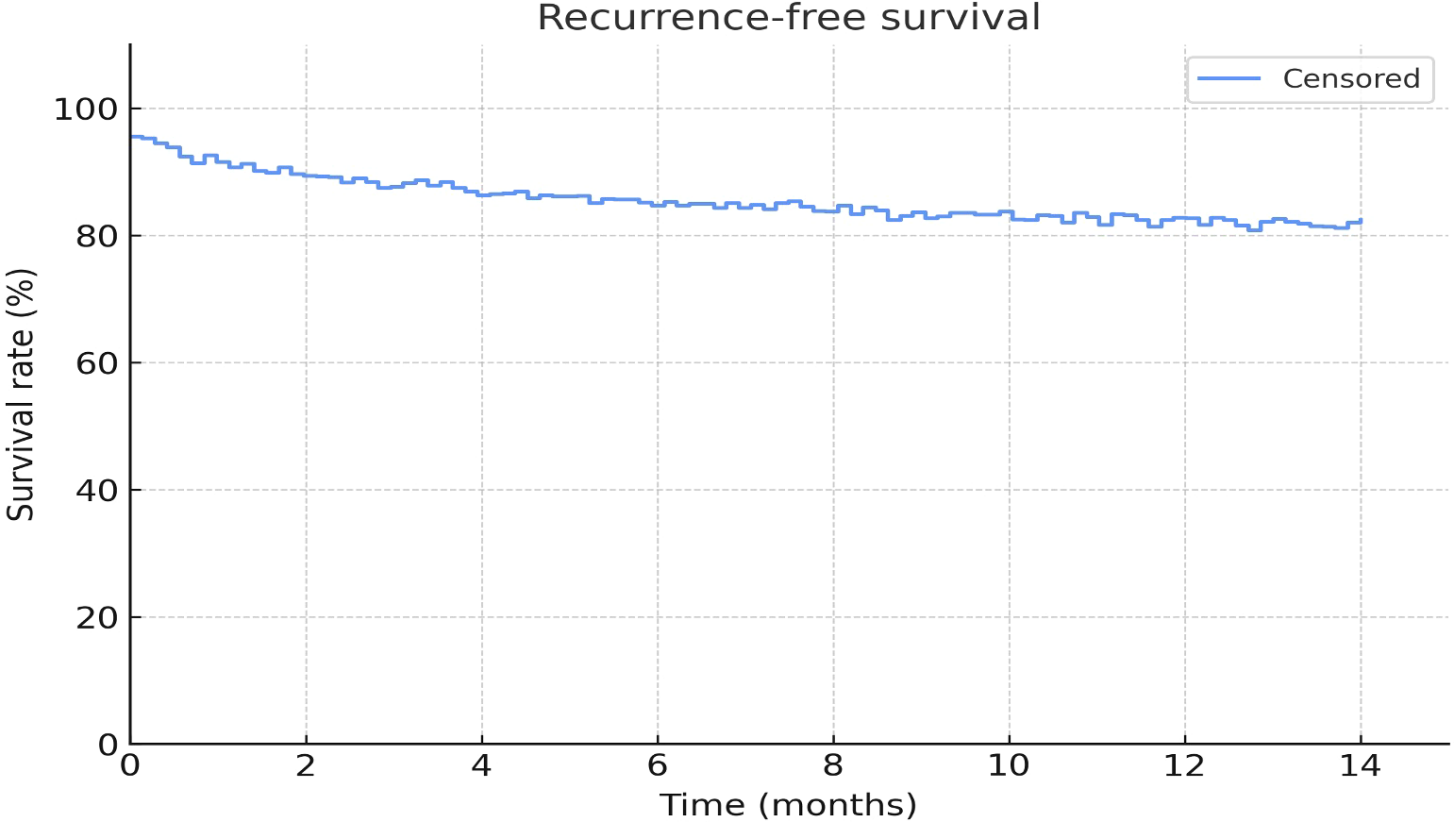
Recurrence-free survival was estimated using the Kaplan–Meier method in the surgery group.

Baseline nodal status was significantly associated with pCR, with higher response rates observed in patients with cN0–1 (50%) disease compared with cN2 (29.7%)(p = 0.031, Fisher’s exact test). However, no independent predictors of pCR were identified in multivariate logistic regression analysis (**Table 3**, **Figure 2**).

**Table 3:** Correlation of selected factors with pCR status.

Characteristics	OR	95%CI	P(Fisher’s exact test)
MRI	0.84	0.36-1.98	0.919
EMVI	0.92	0.39-2.15	0.885
cN0/1 vs. cN2	2.04	1.06–4.90	0.031
cT3 vs. cT4	1.01	0.44–2.31	0.999

The association between clinical complete response (cCR) at restaging and pathological complete response (pCR) is presented in **Table 4**. No significant correlation was observed between cCR and pCR (p = 0.191, Fisher’s exact test).Using pCR as the reference standard, the diagnostic performance of cCR was limited. Clinical complete response demonstrated low sensitivity (15.8%) but high specificity (93.0%) for predicting pCR. The positive predictive value was 60.0%, the negative predictive value was 62.4%, and the overall accuracy was 62.1%.

**Table 4:** Association between clinical complete response and pathological complete response.

Clinical response	pCR	No pCR	Total	P(Fisher’s exact test)
cCR	6 (15.8%)	4 (7.0%)	10	0.191
No cCR	32 (84.2%)	53 (93.0%)	85	
Total	38	57	95	

### Recurrence-free survival

At the time of analysis, 97 patients had undergone curative-intent surgery, with a short follow-up period overall. During follow-up, six patients developed distant metastases, all in the non-pCR group. Kaplan–Meier analysis showed favorable recurrence-free survival, with an estimated 12-month RFS of 93.8% (95% CI 87.8%–99.9%) (**Figure 2**). Median RFS was not reached due to the limited number of events.

### Toxicity

Treatment-related toxicities were common but predominantly grade 1–2 in severity (**Table 5**). Hematologic and gastrointestinal adverse events were the most frequently observed, consistent with the expected toxicity profile of chemoradiotherapy and oxaliplatin-based consolidation. Grade 3–4 events were relatively infrequent, with thrombocytopenia being the most notable severe hematologic toxicity (11.9%). No treatment-related mortality was observed.

**Table 5:** Adverse events (AEs) recorded (N = 99).

Adverse Event	Grade 1–2	Grade 3	Grade 4
Anemia	45 (44.6%)	4 (4%)	1 (1%)
Leukopenia	30 (32.7%)	1 (1%)	–
Thrombocytopenia	46 (45.5%)	9 (8.9%)	3 (3%)
Fatigue	53 (52.5%)	1 (1%)	–
Abdominal pain	40 (39.6%)	1 (1%)	1 (1%)
Diarrhea	31 (30.7%)	2 (2%)	1 (1%)
Nausea/Vomiting	48 (47.5%)	5 (5%)	1 (1%)
Peripheral neuropathy	43 (42.6%)	2 (2%)	–
Elevated liver enzymes	50 (49.5%)	3 (3%)	–

## Discussion

Between June 2023 and April 2024, 101 patients meeting study criteria were enrolled, with a median age of 55 years and a male predominance—findings consistent with RAPIDO and PRODIGE-23 (19, 20). Total neoadjuvant therapy is primarily indicated for patients with locally advanced rectal cancer to achieve maximal tumor downstaging and improve resectability before surgery. In this cohort, only 7.6% of patients were classified as stage II by AJCC 8, all of whom presented with at least cT3N0 disease. A high proportion of patients had cT4 (54.7%) and cN2 (60.4%) disease, compared with 31.8% and 68% in RAPIDO and 18% and 26% in PRODIGE-23, respectively (19, 20). Conversely, the frequency of MRF+ and EMVI+ disease in our study was lower (37.7% and 24.5%) compared with RAPIDO (60% and 32%) and PRODIGE-23 (26% MRF+ only), reflecting the stricter inclusion criteria in those trials, which required high-risk features such as cT4, cN2, EMVI+, MRF+, or enlarged lateral nodes. Tumor location by distance from the anal verge was comparable to PRODIGE-23 (19, 20).

The multimodality management of locally advanced rectal cancer requires integration of radiotherapy, chemotherapy, and surgery to optimize outcomes. Increasingly, TNT has been adopted worldwide based on evidence from recent trials and guidelines, demonstrating improved pCR rates, survival, and quality of life (2, 12). In addition, patients who achieve a complete clinical response may be managed with a nonoperative “watch-and-wait” strategy, thereby avoiding surgical morbidity and preserving function. In our series, 94.1% of patients completed TNT, 72.3% underwent sphincter-preserving surgery, and the pCR rate was 35.7%. Across multiple phase III trials, TNT consistently demonstrated higher pCR rates compared with conventional chemoradiotherapy followed by surgery. In the RAPIDO trial, the pCR rate reached 28% in the experimental arm versus 14**%** in the standard long-course CRT group, despite comparable rates of R0 resection (19). Similarly, the PRODIGE-23 trial demonstrated a pathological complete response rate of 27.5% among patients treated with an induction-based TNT regimen consisting of FOLFIRINOX chemotherapy followed by chemoradiotherapy and total mesorectal excision, compared with 11.7% in the control group receiving standard CRT (20). A recent meta-analysis of TNT versus standard therapy confirmed these findings, indicating a pooled pCR of 28–30% and highlighting the strong predictive value of tumor downstaging for long-term disease control (8, 12). In real-world data, Barba et al. (2025) observed a 30% pCR rate with various TNT sequencing strategies (induction, consolidation, or sandwich) in a single-institution cohort, corroborating the reproducibility of TNT outcomes across clinical settings (9). In addition to these findings, interpretation of TNT outcomes in Vietnam requires consideration of several context-specific factors characteristic of lower-middle-income countries. Vietnam faces substantial disparities in healthcare access, including delayed presentation, variable access to radiotherapy technology, and uneven distribution of trained multidisciplinary oncology teams. These systemic constraints contribute to underdiagnosis of high-risk features, inconsistent staging accuracy, and delayed initiation of neoadjuvant therapy—factors known to adversely influence response rates in rectal cancer. Moreover, socioeconomic barriers such as out-of-pocket treatment costs, logistical challenges in traveling to specialized oncology centers, and disparities between urban and rural populations may affect adherence to long and intensive TNT regimens (21–24).

When compared with international cohorts, patients in Vietnam often present with more advanced disease at baseline and may have undergone prolonged symptom intervals prior to diagnosis due to cultural, educational, and resource-related determinants (22, 25, 26); as in the current study, the high proportion of cT4 and cN2 disease mirrors this epidemiologic tendency. Despite these limitations, TNT delivered in our single-center study achieved a pCR rate of 35.7%—notably higher than rates reported in Western trials such as RAPIDO and PRODIGE-23—suggesting that, when delivered in a specialized high-volume center, TNT can yield outcomes comparable to those in resource-rich settings.

A unique strength of this study is the availability of a robust “internal control” for comparison: historical Vietnamese cohorts treated with conventional long-course CRT followed by surgery and adjuvant chemotherapy have demonstrated substantially lower pCR rates (typically 10–15%), lower treatment completion rates, and higher postoperative complication-related treatment delays. The markedly improved tumor regression and pCR achieved in our TNT cohort thus reflect a meaningful advance over standard care within Vietnam’s oncology landscape (27–30).

The Watch-and-Wait (W&W) strategy is increasingly adopted in patients achieving clinical complete response (cCR) after total neoadjuvant therapy. The pioneering work of Habr-Gama demonstrated that selected patients with cCR managed non-operatively achieved survival outcomes comparable to those with pathologic complete response following surgery (31). Subsequent validation from the International Watch-and-Wait Database confirmed acceptable long-term oncologic outcomes, with most local regrowth events occurring within the first two years and being amenable to salvage surgery (32). More recently, pooled analyses of the OPRA and CAO/ARO/AIO-12 trials demonstrated that a selective W&W strategy after TNT yields disease-free survival comparable to mandatory TME in patients with complete or near-complete response (33). Accurate identification of cCR requires meticulous multimodal assessment, particularly high-resolution MRI combined with endoscopic evaluation, ideally within experienced multidisciplinary centers (34, 35). Therefore, while W&W represents a safe and effective strategy in appropriately selected patients, its implementation must be individualized, taking into account institutional expertise, surveillance capability, and patient preference. In the present study, although 35.7% of patients achieved pathological complete response, W&W was not implemented due to the predefined study protocol requiring surgical resection for pathological assessment. Furthermore, at the time of study initiation, institutional experience with structured W&W surveillance—including standardized MRI reassessment and intensive follow-up—was limited. Nevertheless, based on post-TNT endoscopic and radiologic findings, a subset of patients achieving pCR may retrospectively have fulfilled criteria for nonoperative management. Future prospective studies at our institution will explore the feasibility of a structured W&W protocol in carefully selected low-risk patients.

In our cohort, clinical complete response (cCR) demonstrated high specificity (93.0%) but very low sensitivity (15.8%) for predicting pathological complete response (pCR), with no statistically significant association between the two (p = 0.191). This discrepancy between clinical and pathological response has been consistently described in prior studies. Kokaine et al. reported that although cCR may be observed in a substantial proportion of patients, confirmed pCR rates are considerably lower (36). Similarly, Alexandrescu et al. highlighted the incomplete concordance between cCR and pCR, emphasizing the limitations of clinical assessment in accurately identifying true tumor eradication (37). Imaging-based restaging, particularly MRI, has also demonstrated imperfect correlation with final pathological findings (38). Together, these findings underscore the need for careful interpretation of cCR when considering organ-preservation strategies.

Beyond complete pathological response, overall tumor regression (TRG 0–1) was recorded in 70.4% of patients, suggesting that early intensification of systemic therapy enhances both local tumor eradication and systemic micrometastatic control. In addition to pCR, tumor regression grade (TRG) represents another important histopathologic indicator of response to neoadjuvant therapy. TRG quantifies the degree of replacement of viable tumor cells by fibrosis or inflammatory tissue after treatment and has been shown to correlate strongly with disease-free survival, local recurrence-free survival (LRFS), and overall survival (OS). Patients achieving TRG 0–1 (good responders) generally have a markedly reduced risk of locoregional or distant relapse compared with those showing minimal regression (TRG 2–3) (10). In our study, 70.4% of patients achieved TRG 0–1, aligning with international reports and underscoring that enhanced tumor regression following TNT may serve as a surrogate marker for durable disease control and a potential indicator for organ preservation strategies.

The predictive factors for pCR remain uncertain. Several clinicopathologic variables have been proposed to influence pCR after TNT. High-risk features such as cT4 stage, cN2 nodal disease, EMVI(+), or MRF involvement have been associated with lower pCR probabilities, whereas longer intervals between CRT completion and surgery and the use of consolidation chemotherapy tend to increase pCR likelihood. Consistent with prior reports, nodal stage N2 was an adverse predictor of pCR in our cohort, reflecting the biological aggressiveness of bulky nodal disease observed in the RAPIDO and PRODIGE-23 trials. These findings highlight the role of TNT particularly in patients with high-risk disease (12, 39).

The sequencing of total neoadjuvant therapy may also modulate treatment response. Evidence from the OPRA and CAO/ARO/AIO-12 trials indicates that consolidation chemotherapy administered after chemoradiotherapy yields higher pathological complete response rates than induction-based strategies, likely due to the prolonged cytotoxic exposure of residual tumor cells and enhanced radiosensitization in hypoxic microenvironments. Nevertheless, both induction and consolidation approaches have shown comparable disease-free survival, suggesting that sequencing should be tailored to baseline risk profiles and treatment tolerance (8). Consistent with this paradigm, our study employed a consolidation-based TNT regimen consisting of long-course CRT (50 Gy in 25 fractions with concurrent capecitabine) followed by six cycles of CAPOX or nine cycles of FOLFOX. This sequence achieved a pCR rate of 35.7% and a major response rate (TRG 0–1) of 70.4%, comparable to those reported in RAPIDO (28%) and PRODIGE-23 (27.5%) trials (19, 20). The consolidation phase likely maximized the therapeutic window between CRT and surgery, allowing continuous tumor regression and eradication of micrometastases while maintaining operability. From a biological perspective, consolidation chemotherapy may further exploit radiation-induced vascular remodeling and immunogenic cell death, thereby enhancing drug delivery and cytotoxic immune responses. These synergistic effects contribute to sustained tumor regression and explain the higher proportion of both complete and near-complete responders observed in our cohort (40–43). Accordingly, our findings reinforce the growing consensus that consolidation-based TNT represents a rational and effective strategy for optimizing tumor response while preserving long-term outcomes.

A higher pCR rate has been linked with improved disease-free survival and may allow the implementation of organ-preserving strategies such as *watch-and-wait* in well-selected patients (11, 41). Early results from our cohort showed no local recurrence among complete clinical responders during initial follow-up, supporting TNT as a viable pathway toward rectal preservation. However, consistent with recent literature, longer follow-up is required to confirm durable control and to balance the benefits of organ preservation against potential risks of regrowth.

Multiple randomized trials and meta-analyses have shown that TNT does not increase perioperative morbidity or severe toxicity compared with conventional long-course chemoradiotherapy, despite delivering higher cumulative doses of systemic therapy before surgery. In a systematic review and meta-analysis of seven randomized trials, Kasi et al. reported that TNT improved pCR and DFS without increasing rates of postoperative complications such as anastomotic leak, wound infection, or reoperation compared with standard CRT-based strategies (44). Similarly, long-term results of the CAO/ARO/AIO-12 randomized trial showed no clinically meaningful differences in late toxicity, patient-reported bowel function, or quality of life between induction- and consolidation-type TNT, confirming that intensified neoadjuvant treatment is surgically safe in the long run (45). A recent network meta-analysis by Seow et al. further demonstrated that TNT regimens have comparable rates of grade ≥3 adverse events and surgical complications to conventional LC-CRT, while providing superior systemic control (46). Real-world data from Barba et al. also showed low rates of grade 3–4 toxicity and no treatment-related mortality across different TNT sequencing strategies in a single-institution cohort (9). In our study, 94.1% of patients completed TNT, postoperative outcomes were acceptable with a sphincter-preservation rate of 72.3% and a high R0 resection rate (97.9%), and most adverse events were grade 1–2, with manageable grade 3–4 hematologic toxicity and no treatment-related deaths. These results suggest that, even in a resource-limited setting, TNT can be delivered safely with appropriate monitoring and supportive care. Collectively, both international data and our experience indicate that TNT does not increase surgical risk and is comparable in overall safety to conventional LC-CRT, while offering meaningful improvements in tumor response and downstaging.

Treatment interruptions during long-course chemoradiotherapy (LCRT) represent a clinically relevant issue in locally advanced rectal cancer, as prolongation of neoadjuvant treatment may adversely affect tumor control and survival outcomes. In a retrospective analysis of 299 patients, Lișcu et al. demonstrated significantly worse overall survival among patients experiencing ≥4 days of treatment interruption compared with those with 0–3 days (90.2% vs. 57.9%, p < 0.001), along with inferior disease-free survival and local control (p < 0.001) (47). These findings underscore the importance of maintaining treatment continuity whenever feasible. In this context, total neoadjuvant therapy (TNT), particularly when incorporating short-course radiotherapy (SCRT), may offer practical advantages. The shift from conventional 5–6-week LCRT to a 5-day hypofractionated regimen reduces overall treatment duration and may decrease the likelihood of interruption due to public holidays, machine maintenance, or cumulative acute toxicity. This consideration is especially relevant in resource-constrained settings, where radiotherapy unit capacity is often limited. Although prior meta-analyses have not shown major differences in acute toxicity between LCRT and SCRT, radiation-related adverse effects typically manifest after 1–2 weeks of treatment, suggesting that the condensed SCRT schedule may inherently reduce the risk of interruption (47). Improved adherence to radiotherapy within TNT regimens incorporating SCRT has been noted in recent reports, further supporting its logistical and practical benefits. Together, these considerations suggest that beyond oncologic efficacy, TNT—particularly SCRT-based approaches—may enhance treatment compliance and mitigate the negative impact of radiotherapy interruptions.

Collectively, these findings highlight that TNT is feasible, safe, and effective even within the constraints of a developing healthcare system. They also underscore the need for national guideline updates, investment in radiologic and radiation-oncology infrastructure, and development of trained multidisciplinary teams to ensure broader and equitable access to TNT across Vietnam.

## Conclusion

In summary, total neoadjuvant therapy demonstrated high feasibility, acceptable safety, and strong pathological response rates in this Vietnamese cohort, despite the challenges of a resource-limited healthcare system. We found that our pCR and TRG results aligned with international trial data, meeting the performance benchmarks set by developed countries and high-resource settings, TNT represents a practical and effective strategy for improving rectal cancer care in developing countries. These findings support broader adoption of TNT and the need for updated national guidelines to ensure equitable access across Vietnam.

## Data Availability

The raw data supporting the conclusions of this article will be made available by the authors, without undue reservation.
