# Supplementary material for: Pathologic response after total neoadjuvant therapy in stage II–III rectal cancer: preliminary results from a prospective study from Vietnam
Source: Front Oncol. 2026 Mar 24;16:1774856. doi: 10.3389/fonc.2026.1774856 (PMC13053264; doi:10.3389/fonc.2026.1774856)
Supplement: Supplementary file 2 [file DataSheet2.pdf]

Patient_ID	Year of birth	Age in 2024 (years)	Sex (1=male,2=female)
P001	1976	48	1
P002	1983	41	2
P003	1982	42	1
P004	1979	45	1
P005	1961	63	2
P006	1976	48	2
P007	1964	60	2
P008	1956	68	1
P009	1956	68	2
P010	1975	49	2
P011	1981	43	1
P012	1969	55	1
P013	1964	60	1
P014	1971	53	2
P015	1960	64	2
P016	1977	47	2
P017	1973	51	1
P018	1997	27	1
P019	1959	65	2
P020	1968	56	2
P021	1978	46	2
P022	1967	57	1
P023	1965	59	2
P024	1982	42	1
P025	1952	72	1
P026	1960	64	1
P027	1975	49	1
P028	1978	46	1
P029	1959	65	1
P030	1978	46	2
P031	1976	48	2
P032	1954	70	2
P033	1984	40	2
P034	1970	54	2
P035	1954	70	1
P036	1965	59	1
P037	1982	42	1
P038	1979	45	1
P039	1969	55	1
P040	1958	66	2
P041	1979	45	2
P042	1976	48	1
P043	1956	68	1
P044	1959	65	2
P045	1974	50	1
P046	1985	39	1
P047	1968	56	1
P048	1988	36	1
P049	1963	61	1
P050	1964	60	1
P051	1957	67	1

P052	1960	64	1
P053	1970	54	2
P054	1956	68	2
P055	1966	58	2
P056	1953	71	2
P057	1950	74	2
P058	1980	44	2
P059	1958	66	1
P060	1966	58	1
P061	1967	57	1
P062	1964	60	1
P063	1962	62	1
P064	1983	41	1
P065	1991	33	1
P066	1960	64	1
P067	1956	68	1
P068	1974	50	1
P069	1991	33	1
P070	1955	69	1
P071	1970	54	1
P072	1972	52	2
P073	1963	61	2
P074	1990	34	1
P075	1954	70	2
P076	1972	52	1
P077	1967	57	1
P078	1983	41	1
P079	1980	44	2
P080	1989	35	2
P081	1965	59	2
P082	1976	48	2
P083	1961	63	2
P084	1982	42	2
P085	1966	58	1
P086	1955	69	1
P087	1974	50	1
P088	1974	50	2
P089	1985	39	2
P090	1977	47	1
P091	1980	44	2
P092	1957	67	1
P093	1958	66	1
P094	2002	22	1
P095	1958	66	1
P096	1991	33	1
P097	1969	55	2
P098	1952	72	1
P099	1972	52	1
P100	1967	57	1
P101	1977	47	1

Comorbidities (1=yes,2=no)	Tumor thickness (mm)	Tumor length (mm)
2	15	56
2	10	30
1	17	64
2	12	51
1	19	62
2	13	50
1	19	40
1	28	50
1	14	28
2	13	30
1	9	25
2	14	35
2	28	26
1	13	35
1	27	80
2	16	35
1	17	40
2	10	26
1	16	35
2	19	80
2	10	30
2	12	38
1	16	40
2	12	39
2	28	80
1	11	40
1	11	32
1	20	120
1	17	60
2	13	45
2	12	50
1	11	48
1	10	30
1	20	630
1	20	50
1	20	40
1	17	40
2	18	64
1	19	40
1	21	56
2	21	55
1	14	29
1	13	40
1	15	40
2	11	33
2	14	40
2	16	50
1	14	66
1	10	25
2	14	55
2	13	36

2	20	30
2	15	40
1	30	67
2	17	45
1	14	50
1	12	35
2	18	64
1	20	47
1	13	50
2	17	60
2	10	30
2	16	57
1	15	50
2	13	45
1	18	54
1	10	40
2	15	25
2	30	73
1	20	42
2	9	35
2	14	18
2	15	30
2	20	45
1	12	30
2	23	65
2	9	20
2	20	55
1	11	48
2	8	25
2	14	60
1	17	67
1	24	55
2	15	48
2	19	60
1	15	54
2	20	55
2	12	45
2	13	33
2	14	40
1	18	54
2	12	40
1	24	44
2	16	60
1	15	40
2	30	35
1	12	50
1	15	40
1	12	30
1	17	50
2	13	40

Tumor location by distance from anal verge (mm)
46
30
0
70
8
125
75
108
60
40
140
35
80
56
35
90
50
75
58
108
45
41
120
19
13
65
80
30
45
66
15
95
34
40
55
80
90
96
30
117
100
45
57
90
48
70
120
32
20
35
43

55
95
110
30
80
45
30
30
60
60
110
40
30
16
57
77
60
65
120
50
51
33
30
68
55
52
30
99
103
145
105
40
95
60
102
75
20
123
20
50
70
170
15
16
75
70
55
65
80
115

Tumor location by distance from anal verge (1=lower,2=mid,3=upper rectum)	Clinical T stage (cT)
1	4
1	3
1	3
2	4
1	4
3	4
2	4
3	4
2	3
1	3
3	4
1	3
2	3
2	3
1	3
2	4
2	3
2	3
2	4
3	4
2	3
1	3
3	4
1	3
3	3
2	3
2	4
1	4
2	4
2	4
1	4
2	4
1	3
1	3
2	3
2	3
2	4
2	4
1	3
3	4
2	4
1	3
2	4
2	4
1	3
2	3
3	4
1	4
1	3
1	4
1	3

2	3
2	4
3	4
1	4
3	4
2	4
1	4
1	4
2	4
2	4
3	3
1	4
1	3
1	4
2	4
2	4
2	3
2	4
3	3
2	3
2	4
1	3
1	3
2	4
2	4
2	3
1	4
2	3
3	3
3	4
3	4
1	4
2	4
2	3
2	3
2	4
1	3
3	3
1	3
1	3
2	4
3	3
1	3
2	3
2	4
2	4
2	3
2	4
2	4
3	3

Number of lymph nodes (baseline imaging)	Clinical N stage (cN)	AJCC clinical stage
0	0	IIC
3	1	IIIB
>4	2A	IIIB
3	1	IIIB
>7	2B	IIIC
4	2A	IIIC
	1	IIIB
>4	2A	IIIC
>4	2A	IIIB
0	0	IIA
>4	2A	IIIC
0	0	IIA
>4	2A	IIIB
3	1	IIIB
>4	2A	IIIB
5	2A	IIIC
2	1	IIIB
>4	2A	IIIB
>4	2A	IIIC
2	1	IIIB
4	2A	IIIB
4	2A	IIIB
5	2A	IIIC
2	1	IIIB
0	0	IIA
0	0	IIA
>4	2A	IIIB
>7	2B	IIIC
>4	2A	IIIC
>7	2B	IIIC
5	2A	IIIC
>7	2B	IIIC
3	1	IIIB
3	1	IIIB
>4	2A	IIIB
>4	2A	IIIB
>4	2A	IIIC
>4	2A	IIIC
>4	2A	IIIB
>7	2B	IIIC
>7	2B	IIIC
>4	2A	IIIB
3	1	IIIB
6	2A	IIIC
3	1	IIIB
4	2A	IIIB
>7	2B	IIIC
>7	2B	IIIC
2	1	IIIB
>7	2B	IIIC
3	1	IIIB

	4	2A		IIIB
>4		2A		IIIC
>7		2B		IIIC
	4	2A		IIIC
>4		2A		IIIC
>4		2A		IIIC
	4	2A		IIIC
>7		2B		IIIC
	7	2B		IIIC
>7		2B		IIIC
	3		1	IIIB
>7		2B		IIIC
>7		2B		IIIC
	5	2A		IIIC
>4		2A		IIIC
	2		1	IIIB
	2		1	IIIB
>7		2B		IIIC
	2		1	IIIB
	3		1	IIIB
	0		0	IIB
	3		1	IIIB
>4		2A		IIIB
	0		0	IIB
>7		2B		IIIC
	1		1	IIIB
	3		1	IIIB
	3		1	IIIB
	2		1	IIIB
	4	2A		IIIC
>4		2A		IIIC
>7		2B		IIIC
>7		2B		IIIC
	2		1	IIIB
>4		2A		IIIB
>7		2B		IIIC
	3		1	IIIB
>4			1	IIIB
	5	2A		IIIB
	0		0	IIA
>7		2B		IIIC
	5	2A		IIIB
>4		2A		IIIB
			1	IIIB
	0		0	IIB
>4		2A		IIIC
	5	2A		IIIB
	3		1	IIIB
	5	2A		IIIC
	4	2A		IIIB

Suspicious extracapsular nodal extension (imaging)(1=yes,2=no,3= no data available)
2
2
2
2
2
2
2
2
2
2
2
2
2
2
2
2
2
2
2
2
2
2
2
2
2
2
2
2
2
2
2
2
2
2
2
2
2
2
2
2
2
2
2
2
2
2
2
2
2
2
2

2
2
2
2
2
3
2
2
2
2
2
2
2
2
2
2
2
2
2
2
2
2
2
3
2
2
2
2
2
2
2
2
2
2
2
2
3
2
3
2
2
2
2
2
2
2
2
2
2
2

Mesorectal fascia involvement (MRF, 1–3)(1=yes,2=no,3= no data available)
1
1
3
2
1
2
1
2
2
3
2
1
2
2
1
2
2
2
2
3
2
2
1
2
2
2
1
1
1
1
1
2
3
3
2
1
2
2
1
3
2
2
1
1
2
2
3
1
3
1
3

2
3
3
1
3
2
1
1
3
1
2
2
1
2
1
3
1
1
2
2
2
3
1
1
2
2
1
2
2
3
3
1
1
2
2
3
1
1
2
2
2
1
3
3
2
1
2
2
1
3

Extramural vascular invasion (EMVI, 1–3)(1=yes,2=no,3= no data available)
2
2
3
2
2
2
1
1
2
3
2
2
3
2
2
2
2
3
3
1
2
3
1
2
2
2
1
1
3
1
1
3
2
3
3
1
1
2
2
3
1
2
2
2
2
2
1
1
2
1
2

2
1
3
1
2
2
1
2
3
1
2
1
2
2
1
3
2
1
2
2
2
3
2
2
1
2
2
1
2
2
3
1
1
2
2
3
2
3
2
2
3
1
2
3
1
3
2
1
2
1

Invasion of pelvic wall/adjacent organ (1=yes, 0= no)
1
0
0
0
1
0
0
0
0
0
0
0
0
0
0
0
0
0
0
0
0
0
0
0
0
0
0
1
0
0
0
0
0
0
0
0
0
1
0
0
0
0
0
0
0
0
0
0
0
0
0

0
0
0
1
0
0
1
1
0
0
0
0
0
1
1
0
0
0
0
0
0
0
0
0
0
0
0
0
0
0
0
1
0
0
0
0
0
0
0
0
0
0
0
0
0
0
0
0
0
0

Histologic grade before treatment (1=well,2=moderate,3=poorly differentiated)
2
2
2
2
2
2
2
2
2
2
1
2
2
2
2
1
2
2
2
2
2
2
2
2
2
2
2
2
2
3
2
2
2
2
2
2
2
2
2
1
2
2
2
2
2
2
2
2
2
2
2

2
2
2
3
2
2
2
2
2
2
2
2
1
2
2
2
2
2
2
2
2
2
2
2
2
2
2
2
2
2
2
2
2
2
2
2
1
2
2
2
2
2
3
2
2
2
2
2
2
2

Tumor response after chemoradiotherapy (1–4)(1=CR,2=PR,3=SD,4=PD)	yTx	yNx	ycAJCCx
2	4A	0	IIB
3	3	1	IIIB
2	3	0	IIA
2	4A	0	IIB
2	3	0	IIA
2	4A	0	IIB
3	3	1	IIIB
3	4A	1	IIIB
2	3	0	IIA
3	3	0	IIA
2	3	0	IIA
3	3	0	IIA
2	3	0	IIA
2	3	0	IIA
3	3	1	IIIB
3	4A	1	IIIB
3	3	1	IIIB
2	3	0	IIA
2	3	0	IIA
2	4A	0	IIA
2	3	1	IIIB
2	3	0	IIA
3	3	1	IIIB
2	3	0	IIA
2	2	0	I
3	3	0	IIA
3	3	1	IIIB
3	4B	1	IIIC
3	3	1	IIIB
2	3	0	IIA
3	3	1	IIIB
2	3	0	IIA
2	3	0	IIA
3	3	1	IIIB
2	3	0	IIA
3	3	1	IIIB
2	2	0	I
3	4B	1	IIIC
2	3	0	IIA
2	4B	0	IIC
2	3	0	IIA
3	3	1	IIIB
2	3	0	IIA
3	4A	1	IIIB
3	3	1	IIIB
2	1	0	I
2	3	0	IIA
2	4A	0	IIB
2	2	1	IIIA
3	3	1	IIIB
3	3	1	IIIB

2	3	0	IIA
2	3	0	IIA
2	1	0	I
2	4B	0	IIC
3	3	1	IIIB
3	3	1	IIIB
2	4B	0	IIC
3	3	1	IIIB
2	3	0	IIA
2	4A	0	IIB
2	3	0	IIA
3	4A	1	IIIB
2	2	0	I
3	4B	1	IIIC
3	4	2	IIIC
2	4A	0	IIB
2	3	0	IIA
3	4A	0	IIB
2	1	1	IIIA
2	2	0	I
3	4A	0	IIB
2	2	0	I
3	4	1	IIIB
3	4	0	IIB
2	4A	0	IIB
2	3	0	IIA
3	3	1	IIIB
2	3	0	IIA
2	3	0	IIA
3	4A	0	IIB
3	4	0	IIB
3	4B	0	IIC
2	4A	0	IIB
2	2	0	I
3	3	1	IIIB
3	4	2	IIIC
2	3	0	IIA
2	3	0	IIA
2	3	0	IIA
3	3	0	IIA
2	3	0	IIA
2	4A	0	IIB
3	4A	2	IIIC
2	3	0	IIA
3	4A	0	IIB
2	4A	0	IIB
2	3	0	IIA
2	4A	0	IIB
3	4	1	IIIB
2	3	0	IIA

Disease progression (1=yes,2=no)	Site of progression (1=local,2=distant)
2	
2	
2	
2	
2	
2	
2	
2	
2	
2	
2	
2	
2	
2	
2	
2	
2	
2	
2	
2	
2	
2	
2	
2	
2	
2	
2	
2	
2	
2	
2	
2	
2	
2	
2	
2	
2	
2	
2	
2	
2	
2	
2	
2	
2	
2	
2	
2	
2	
2	
2	

2
2
2
2
2
2
2
2
2
2
2
2
2
2
2
2
2
2
2
2
2
2
2
2
2
2
2
2
2
2
2
2
2
2
2
2
2
2
2
2
2
2
2
2
2
2
2
2
2
2

Tumor response after chemotherapy (1–4)(1=CR,2=PR,3=SD,4=PD)	yTc	yNc	ycAJCCc
2	3	0	IIA
3	3	1	IIIB
4	3	2	IIIB
2	3	0	IIA
3	3	0	IIA
2	1	0	I
2	3	0	IIA
3	4	1	IIIB
3	3	0	IIA
1	0	0	0
3	3	0	IIA
3	3	0	IIA
2	3	0	IIA
3	3	0	IIA
2	3	0	IIA
2	4	0	IIB
2	3	0	IIA
3	3	0	IIA
3	3	0	IIA
4	4	0	IIB
2	1	0	I
3	3	0	IIA
3	3	1	IIIB
3	3	0	IIA
4	3	0	IIA
3	3	0	IIA
2	2	0	I
2	4	0	IIB
3	3	1	IIIB
4	4	0	IIB
3	3	1	IIIB
2	2	0	I
3	3	0	IIA
1	0	0	0
1	0	0	0
3	3	1	IIIB
3	2	0	I
2	4	0	IIB
3	3	0	IIA
2	3	0	IIA
2	2	0	I
2	3	0	IIA
3	3	0	IIA
3	4	1	IIIB
1	0	0	0
3	1	0	I
4	4	1	IIIB
2	3	0	IIA
2	2	0	I
2	3	0	IIA
2	3	0	IIA

4	3	1	IIIB	
3	3	0	IIA	
3	2	0	I	
2	4	0	IIB	
2	3	0	IIA	
2	3	0	IIA	
4	4	2	IIIC	
2	3	0	IIA	
3	3	0	IIA	
3	4	0	IIB	
3	3	0	IIA	
3	4	1	IIIB	
3	2	0	I	
3	4	1	IIIC	
2	3	0	IIA	
3	4	0	IIB	
3	3	0	IIA	
4	3	1	IIIB	
1	0	0		0
3	2	0	I	
2	2	0	I	
1	0	0		0
2	3	0	IIA	
1	0	0		0
3	4	0	IIB	
1	0	0		0
2	4	0	IIB	
3	3	0	IIA	
2	2	0	I	
4	4	1	IIIB	
2	3	0	IIA	
2	4	0	IIB	
2	2	0	I	
3	2	0	I	
2	3	0	IIA	
2	4	0	IIB	
3	3	0	IIA	
3	3	0	IIA	
3	3	0	IIA	
3	3	0	IIA	
3	3	0	IIA	
1	0	0		0
3	4	2	IIIC	
3	3	0	IIA	
4	4	1	IV	
4	4	1	IV	
4	3	0	IV	
4	4	0	IV	
1	0	0		0
1	0	0		0

Disease progression during chemotherapy (1=yes,2=no)
2
2
2
2
2
2
2
2
2
2
2
2
2
2
2
2
2
2
2
2
2
2
2
2
2
2
2
2
2
2
2
2
2
2
2
2
2
2
2
2
2
2
2
2
2
2
2
2
2

2
2
2
2
2
2
2
2
2
2
2
2
2
2
2
2
2
2
2
2
2
2
2
2
2
2
2
2
2
2
2
2
2
2
2
2
2
2
2
2
2
2
2
1
1
1
1
1
1
2

Site of progression during chemotherapy (1=local,2=distant)	Distant metastasis site

2	liver, peritoneum, lymph node
2	Lung
2	Lung
2	Lung
1	

Endoscopic assessment (1=residual tumor,2=no visible tumor)
1
2
1
1
1
1
1
1
2
2
2
2
2
1
1
1
2
2
2
1
2
1
1
1
2
1
2
1
1
1
2
2
2
1
2
1
1
2
1
2
1
1
1
1
1
1
1
1
1
2
2

1
2
1
1
1
1
1
1
2
1
1
1
1
1
2
2
1
1
2
1
1
2
1
1
1
1
1
1
2
1
1
1
1
1
1
1
1
1
1
1
1
2
1
1
1
1
1
1
2
2

Overall response to total neoadjuvant therapy (1=CR,2=PR,3=SD,4=PD)
2
3
3
2
2
2
2
3
2
1
2
3
2
2
2
1
2
2
2
2
2
2
3
2
3
3
2
2
3
2
3
2
2
1
1
3
2
2
2
2
2
2
2
3
1
2
3
2
2
2
2

3
2
2
2
2
2
3
2
2
2
2
3
2
3
2
2
2
3
1
2
2
1
2
1
2
1
2
2
2
3
2
2
2
2
2
2
2
2
2
3
2
1
3
2
4
4
4
4
1
2

Sphincter preservation (1=yes,2=no)	Residual tumor status (0=R0,1=R1,2=R2)	Pathologic T stage (ypT)
1	0	3
1	0	0
2	0	0
1	0	2
2	0	3
1	0	3
1	0	3
1	0	3
1	0	0
2	0	2
1	0	0
1	0	0
1	0	0
1	0	3
1	0	0
1	0	0
1	0	4
1	0	0
1	0	0
1	0	0
1	0	1
2	0	1
1	0	2
2	0	3
1	0	1
1	0	3
1	0	0
2	0	3
2	0	0
1	0	0
2	0	2
1	0	0
2	0	0
1	0	0
1	0	0
1	0	1
1	0	3
1	0	0
2	0	2
1	0	0
1	0	2
1	0	0
1	0	2
1	0	0
1	0	2
1	0	1
1	0	3
2	0	2
1	0	2
2	0	2
1	0	0

1	0	2
1	0	0
1	0	0
2	0	0
1	0	2
1	0	3
2	0	3
1	0	3
1	0	2
1	0	0
1	0	2
1	0	0
1	0	3
2	0	0
1	0	0
1	0	3
1	0	2
1	0	0
1	0	0
2	0	2
1	0	2
1	0	0
2	0	3
1	0	3
1	2	0
1	0	0
2	0	4
1	0	0
1	0	0
1	0	0
1	0	2
2	0	0
1	0	3
1	0	3
1	0	0
1	0	3
2	0	0
1	0	0
2	0	3
1	0	2
1	0	0
1	0	4
2	2	0
1	0	3

The patient refused surgery
The patient refused surgery

Pathologic N stage (ypN)	Pathologic M stage (ypM)	Pathologic AJCC stage (ypAJCC)
0	0	IIA
0	0	0
0	0	0
0	0	I
0	0	IIA
0	0	IIA
1	0	IIIB
1	0	IIIB
0	0	0
0	0	I
0	0	0
0	0	0
0	0	0
0	0	IIA
0	0	0
0	0	0
0	0	IIB
0	0	0
0	0	0
0	0	IIB
1	0	IIIA
0	0	I
0	0	I
0	0	IIA
0	0	I
0	0	IIA
1	0	IIIA
0	0	IIA
0	0	0
0	0	0
0	0	I
0	0	0
0	0	0
0	0	0
0	0	0
0	0	I
0	0	IIA
0	0	0
0	0	I
0	0	0
0	0	I
0	0	0
0	0	I
1	0	IIIA
1	0	IIIA
0	0	I
0	0	IIA
0	0	I
0	0	I
0	0	I
0	0	0

1	0	IIIA	
0	0		0
0	0		0
0	0		0
0	0	I	
0	0	IIA	
2	0	IIIB	
0	0	IIA	
0	0	I	
0	0		0
0	0	I	
2	0	IIIC	
0	0	IIA	
0	0		0
0	0		0
0	0	IIA	
0	0	I	
0	0		0
0	0		0
0	0	I	
1	0	IIIA	
0	0		0
0	0	IIA	
0	0	IIA	
1	0	IV	
0	0		0
0	0	IIB	
0	0		0
0	0		0
0	0		0
0	0	I	
0	0		0
0	0	IIA	
0	0	IIA	
0	0		0
0	0	IIA	
0	0		0
0	0		0
0	0	IIA	
0	0	I	
0	0		0
0	0	IIB	
2	1	IV	
0	1	IV	

Lymphovascular invasion (LVI, pathology)(0=No data, 1=yes, 2=no)
0
2
2
2
2
2
1
0
2
0
0
0
2
0
0
2
0
0
2
2
1
2
2
2
2
1
2
0
2
0
0
2
0
0
0
0
2
2
2
2
0
2
0
2
2
2
2
2
2
2
2

2
0
2
0
0
2
2
2
2
2
3
0
2
1
2
0
0
1
2
0
0
2
1
0
2
2
2
0
2
0
0
2
2
0
2
2
0
2
0
0
2
2
2
2

Perineural invasion (PNI, pathology)(0=No data, 1=yes, 2=no)
0
2
2
1
2
2
2
0
2
0
0
0
2
0
0
1
0
0
2
2
2
2
2
2
2
2
2
0
2
0
0
2
0
0
0
0
2
2
2
2
0
2
0
2
2
2
2
2
2
1
2

2
0
2
0
0
2
2
2
2
2
3
0
2
1
2
0
0
1
2
0
0
2
2
0
2
1
1
0
2
0
0
2
2
0
2
2
0
2
0
0
2
2
2
2

Pathologic tumor regression grade (TRG, 0=pCR,1=near-complete,2=partial,3=poor)
0
1
2
3
1
1
3
0
1
0
0
0
2
0
0
2
0
0
1
2
1
2
2
1
1
3
1
0
1
0
0
1
0
0
0
0
1
2
2
2
0
1
0
1
3
1
1
1
2
1
1

1
0
1
0
0
3
1
1
2
3
1
0
2
3
1
0
0
1
1
0
0
1
2
0
2
2
2
0
1
0
0
1
2
0
3
2
0
1
0
0
2
1
3
1

Histologic grade after treatment (1=well,2=moderate,3=poor,4=no residual tumor)
2
4
4
2
2
1
2
2
4
2
4
4
4
2
4
4
2
4
4
2
2
2
3
2
2
2
2
2
4
4
2
4
4
4
4
2
2
2
2
4
2
4
2
2
2
2
2
2
2
1
4

2
4
4
3
2
2
3
2
2
4
1
2
2
4
4
2
2
4
4
2
2
4
2
2
2
4
2
4
4
2
2
4
2
2
4
2
4
4
2
2
2
2
3
2

Postoperative complications (1=yes,2=no)	Postoperative length of stay (days)
2	7
2	6
2	6
2	8
2	6
2	6
2	6
1	7
2	7
2	8
2	7
2	6
2	6
2	7
2	7
2	7
1	7
2	7
2	7
2	7
2	7
2	4
2	7
2	7
2	7
2	7
1	8
1	7
2	7
2	4
2	6
2	6
2	6
2	6
2	10
2	7
1	6
1	14
2	7
2	7
2	7
2	6
2	6
2	7
2	7
2	7
2	7
2	6
2	4
2	7
1	32

1	8
2	7
2	6
2	6
1	7
2	7
2	6
2	7
2	9
1	7
2	8
2	5
1	
2	7
1	20
2	7
2	6
2	7
2	7
2	6
1	7
2	7
2	7
2	10
2	7
2	6
2	7
2	
2	7
2	8
2	7
2	6
2	9
2	7
2	4
2	7
2	7
2	7
2	7
2	7
2	7
2	6
2	7
1	18

Date of start of follow-up	Date of surgery	Disease-free survival follow-up (status)
2023-10-01 00:00:00	2024-03-12 00:00:00	2025-10-31 00:00:00
2024-03-04 00:00:00	2024-07-12 00:00:00	2025-10-31 00:00:00
2024-05-21 00:00:00	2025-03-13 00:00:00	2025-10-31 00:00:00
2024-03-20 00:00:00	2024-12-10 00:00:00	2025-10-31 00:00:00
2024-01-31 00:00:00	2025-02-07 00:00:00	2025-10-31 00:00:00
2024-01-23 00:00:00	2024-02-07 00:00:00	2025-10-31 00:00:00
2023-11-24 00:00:00	2024-07-13 00:00:00	2025-10-31 00:00:00
2024-03-25 00:00:00	2025-01-17 00:00:00	2025-10-31 00:00:00
2023-11-14 00:00:00	2024-08-01 00:00:00	2025-10-31 00:00:00
2024-01-03 00:00:00	2024-09-28 00:00:00	2025-10-31 00:00:00
2024-07-22 00:00:00	2025-04-10 00:00:00	2025-10-31 00:00:00
2024-06-06 00:00:00	2025-03-25 00:00:00	2025-10-31 00:00:00
2024-01-19 00:00:00	2024-11-05 00:00:00	2025-10-31 00:00:00
2024-06-20 00:00:00	2025-03-19 00:00:00	2025-10-31 00:00:00
2024-04-15 00:00:00	2025-01-13 00:00:00	2025-10-31 00:00:00
2024-04-15 00:00:00	2024-12-03 00:00:00	2025-10-31 00:00:00
2023-12-14 00:00:00	2024-10-16 00:00:00	2025-10-31 00:00:00
2024-05-06 00:00:00	2025-02-11 00:00:00	2025-10-31 00:00:00
2024-06-17 00:00:00	2025-02-20 00:00:00	2025-10-31 00:00:00
2023-11-29 00:00:00	2024-10-17 00:00:00	2025-10-31 00:00:00
2024-05-09 00:00:00	2025-02-20 00:00:00	2025-10-31 00:00:00
2024-06-20 00:00:00	2025-03-03 00:00:00	2025-10-31 00:00:00
2023-11-01 00:00:00	2024-09-12 00:00:00	2025-10-31 00:00:00
2023-12-21 00:00:00	2024-11-07 00:00:00	2025-10-31 00:00:00
2024-01-09 00:00:00	2024-10-08 00:00:00	2025-10-31 00:00:00
2024-02-15 00:00:00	2024-11-08 00:00:00	2025-10-31 00:00:00
2024-08-01 00:00:00	2025-04-05 00:00:00	2025-10-31 00:00:00
2024-04-20 00:00:00	2025-04-01 00:00:00	2025-10-31 00:00:00
2024-06-17 00:00:00	2025-05-22 00:00:00	2025-10-31 00:00:00
2023-08-24 00:00:00	2024-06-20 00:00:00	2025-10-31 00:00:00
2024-05-06 00:00:00	2025-01-09 00:00:00	2025-12-05 00:00:00
2024-02-26 00:00:00	2024-11-22 00:00:00	2025-10-31 00:00:00
2023-10-05 00:00:00	2024-08-22 00:00:00	2025-10-31 00:00:00
2023-05-12 00:00:00	2024-06-28 00:00:00	2025-10-31 00:00:00
2024-06-10 00:00:00	2025-04-05 00:00:00	2025-10-31 00:00:00
2024-05-10 00:00:00	2025-02-19 00:00:00	2025-10-31 00:00:00
2024-03-19 00:00:00	2024-11-23 00:00:00	2025-10-31 00:00:00
2024-06-13 00:00:00	2025-03-20 00:00:00	2025-10-31 00:00:00
2024-07-29 00:00:00	2025-05-12 00:00:00	2025-10-31 00:00:00
2024-02-19 00:00:00	2024-12-04 00:00:00	2025-10-31 00:00:00
2023-11-24 00:00:00	2024-09-25 00:00:00	2025-10-31 00:00:00
2024-05-03 00:00:00	2025-01-16 00:00:00	2025-10-31 00:00:00
2023-11-17 00:00:00	2024-08-31 00:00:00	2025-10-31 00:00:00
2024-02-22 00:00:00	2024-12-17 00:00:00	2025-10-31 00:00:00
2023-10-11 00:00:00	2024-06-20 00:00:00	2025-10-31 00:00:00
2023-12-25 00:00:00	2024-08-30 00:00:00	2025-10-31 00:00:00
2023-11-15 00:00:00	2024-08-08 00:00:00	2025-10-31 00:00:00
2024-03-13 00:00:00	2024-12-12 00:00:00	2025-10-31 00:00:00
2024-06-06 00:00:00	2025-03-07 00:00:00	2025-10-31 00:00:00
2024-06-05 00:00:00	2025-03-19 00:00:00	2025-10-31 00:00:00
2023-11-28 00:00:00	2024-10-04 00:00:00	2025-10-31 00:00:00

2024-06-14 00:00:00	2025-05-05 00:00:00	2025-10-31 00:00:00
2023-11-13 00:00:00	2024-07-08 00:00:00	2025-10-31 00:00:00
2024-01-09 00:00:00	2025-01-03 00:00:00	2025-10-31 00:00:00
2023-10-20 00:00:00	2024-10-03 00:00:00	2025-10-31 00:00:00
2024-03-20 00:00:00	2024-12-19 00:00:00	2025-10-31 00:00:00
2024-05-15 00:00:00	2025-03-10 00:00:00	2025-10-31 00:00:00
2023-09-14 00:00:00	2024-07-10 00:00:00	2025-06-24 00:00:00
2023-10-24 00:00:00	2024-09-25 00:00:00	2025-10-31 00:00:00
2023-11-13 00:00:00	2024-05-25 00:00:00	2025-10-31 00:00:00
2024-05-17 00:00:00	2025-01-21 00:00:00	2025-10-31 00:00:00
2024-03-26 00:00:00	2024-11-28 00:00:00	2025-10-31 00:00:00
2024-06-24 00:00:00	2025-01-21 00:00:00	2025-10-31 00:00:00
2024-01-12 00:00:00	2024-10-07 00:00:00	2025-10-31 00:00:00
2024-01-04 00:00:00	2025-03-10 00:00:00	2025-10-31 00:00:00
2023-12-04 00:00:00	2024-09-19 00:00:00	2025-10-31 00:00:00
2025-03-01 00:00:00	2024-12-16 00:00:00	2025-10-31 00:00:00
2024-02-29 00:00:00	2024-11-02 00:00:00	2025-10-31 00:00:00
2023-12-08 00:00:00	2024-10-08 00:00:00	2025-10-31 00:00:00
2024-01-24 00:00:00	2024-12-03 00:00:00	2025-10-31 00:00:00
2024-01-29 00:00:00	2024-11-07 00:00:00	2025-10-31 00:00:00
2024-05-07 00:00:00	2025-01-07 00:00:00	2025-10-31 00:00:00
2024-01-15 00:00:00	2024-12-23 00:00:00	2025-10-31 00:00:00
2024-06-12 00:00:00	2025-04-02 00:00:00	2025-10-31 00:00:00
2024-01-12 00:00:00	2024-11-05 00:00:00	2025-10-31 00:00:00
2024-02-27 00:00:00	2024-12-23 00:00:00	2025-01-14 00:00:00
2024-02-20 00:00:00	2024-11-02 00:00:00	2025-10-31 00:00:00
2023-12-04 00:00:00	2024-09-11 00:00:00	2024-12-06 00:00:00
2024-02-02 00:00:00	2024-11-08 00:00:00	2025-02-11 00:00:00
2024-01-25 00:00:00	2024-11-14 00:00:00	2025-10-31 00:00:00
2024-01-18 00:00:00	2024-11-18 00:00:00	2025-10-31 00:00:00
2024-03-29 00:00:00	2024-11-27 00:00:00	2025-10-31 00:00:00
2024-07-08 00:00:00	2025-03-29 00:00:00	2025-10-31 00:00:00
2024-02-28 00:00:00	2024-10-23 00:00:00	2025-05-23 00:00:00
2023-12-08 00:00:00	2024-08-10 00:00:00	2025-10-31 00:00:00
2024-02-26 00:00:00	2024-12-18 00:00:00	2025-10-31 00:00:00
2024-07-16 00:00:00	2025-03-17 00:00:00	2025-10-31 00:00:00
2024-02-12 00:00:00	2024-12-26 00:00:00	2025-10-31 00:00:00
2024-06-12 00:00:00	2025-02-25 00:00:00	2025-10-31 00:00:00
2024-02-27 00:00:00	2025-01-14 00:00:00	2025-05-19 00:00:00
2023-09-08 00:00:00	2024-08-12 00:00:00	2025-10-31 00:00:00
2024-01-24 00:00:00	2024-11-13 00:00:00	2025-10-31 00:00:00
2024-01-16 00:00:00	2024-09-24 00:00:00	2025-10-31 00:00:00
2024-01-10 00:00:00	2024-09-27 00:00:00	2024-09-27 00:00:00
2024-01-13 00:00:00	2024-12-09 00:00:00	2025-03-25 00:00:00
2023-10-12 00:00:00		2024-06-26 00:00:00
2024-01-22 00:00:00		2024-09-09 00:00:00
2024-02-01 00:00:00		2024-09-23 00:00:00
2024-05-27 00:00:00		2025-01-13 00:00:00
2024-01-30 00:00:00		2025-10-31 00:00:00
2024-06-28 00:00:00		2025-10-31 00:00:00

Disease-free survival (months)
19
15
7
10
8
20
15
9
14
13
6
7
11
7
9
10
12
8
8
12
8
7
13
11
12
11
6
6
5
16
10
11
14
16
6
8
11
7
5
10
13
9
14
10
16
14
14
10
7
7
12

5
15
9
12
10
7
11
13
17
9
11
9
12
7
13
10
11
12
10
11
9
10
6
11
0
11
2
3
11
11
11
7
7
14
10
7
10
8
4
14
11
13
0
3

Disease-free status (1=no recurrence,2=local recurrence,3=distant metastasis)
1
1
1
1
1
1
1
1
1
1
1
1
1
1
1
1
1
1
1
1
1
1
1
1
1
1
1
1
1
1
3
1
1
1
1
1
1
1
1
1
1
1
1
1
1
1
1
1
1
1
1

1
1
1
1
1
1
2
1
1
1
1
1
1
1
1
1
1
1
1
1
1
1
1
1
3
1
3
3
1
1
1
1
3
1
1
1
1
1
3
1
1
1
3
3
3
3
3
3
2
1

Site of distant metastasis	Overall survival follow-up (status)	Overall survival (months)
	2025-10-31 00:00:00	24
	2025-10-31 00:00:00	19
	2025-10-31 00:00:00	17
	2025-10-31 00:00:00	19
	2025-10-31 00:00:00	21
	2025-10-31 00:00:00	21
	2025-10-31 00:00:00	23
	2025-10-31 00:00:00	19
	2025-10-31 00:00:00	23
	2025-10-31 00:00:00	21
	2025-10-31 00:00:00	15
	2025-10-31 00:00:00	16
	2025-10-31 00:00:00	21
	2025-10-31 00:00:00	16
	2025-10-31 00:00:00	18
	2025-10-31 00:00:00	18
	2025-10-31 00:00:00	22
	2025-10-31 00:00:00	17
	2025-10-31 00:00:00	16
	2025-10-31 00:00:00	23
	2025-10-31 00:00:00	17
	2025-10-31 00:00:00	16
	2025-10-31 00:00:00	23
	2025-10-31 00:00:00	22
	2025-10-31 00:00:00	21
	2025-10-31 00:00:00	20
	2025-10-31 00:00:00	14
	2025-10-31 00:00:00	18
	2025-10-31 00:00:00	16
	2025-10-31 00:00:00	26
Pulmonary nodules and peritoneal	2025-10-31 00:00:00	17
	2025-10-31 00:00:00	20
	2025-10-31 00:00:00	24
	2025-10-31 00:00:00	29
	2025-10-31 00:00:00	16
	2025-10-31 00:00:00	17
	2025-10-31 00:00:00	19
	2025-10-31 00:00:00	16
	2025-10-31 00:00:00	15
	2025-10-31 00:00:00	20
	2025-10-31 00:00:00	23
	2025-10-31 00:00:00	17
	2025-10-31 00:00:00	23
	2025-10-31 00:00:00	20
	2025-10-31 00:00:00	24
	2025-10-31 00:00:00	22
	2025-10-31 00:00:00	23
	2025-10-31 00:00:00	19
	2025-10-31 00:00:00	16
	2025-10-31 00:00:00	16
	2025-10-31 00:00:00	23

	2025-10-31 00:00:00	16
	2025-10-31 00:00:00	23
	2025-10-31 00:00:00	21
	2025-10-31 00:00:00	24
	2025-10-31 00:00:00	19
	2025-10-31 00:00:00	17
	2025-10-31 00:00:00	25
	2025-10-31 00:00:00	24
	2025-10-31 00:00:00	23
	2025-10-31 00:00:00	17
	2025-10-31 00:00:00	19
	2025-10-31 00:00:00	16
	2025-10-31 00:00:00	21
	2025-10-31 00:00:00	21
	2025-10-31 00:00:00	22
	2025-10-31 00:00:00	7
	2025-10-31 00:00:00	20
	2025-10-31 00:00:00	22
	2025-10-31 00:00:00	21
	2025-10-31 00:00:00	21
	2025-10-31 00:00:00	17
	2025-10-31 00:00:00	21
	2025-10-31 00:00:00	16
	2025-10-31 00:00:00	21
liver	2025-10-31 00:00:00	20
	2025-10-31 00:00:00	20
Bone	2025-10-31 00:00:00	22
Peritoneum and lung	2025-10-31 00:00:00	20
	2025-10-31 00:00:00	21
	2025-10-31 00:00:00	21
	2025-10-31 00:00:00	19
	2025-10-31 00:00:00	15
Liver and ovary	2025-10-31 00:00:00	20
	2025-10-31 00:00:00	22
	2025-10-31 00:00:00	20
	2025-10-31 00:00:00	15
	2025-10-31 00:00:00	20
	2025-10-31 00:00:00	16
Lung and liver	2025-10-31 00:00:00	20
	2025-10-31 00:00:00	25
	2025-10-31 00:00:00	21
	2025-10-31 00:00:00	21
Pelvic peritoneum (intraoperative finding)	2025-10-31 00:00:00	21
Liver and lung	2025-10-31 00:00:00	21
Liver, peritoneum, lymph nodes	2025-10-31 00:00:00	24
Lung	2025-10-31 00:00:00	21
Lung	2025-10-31 00:00:00	20
Lung	2025-10-31 00:00:00	17
Local regrowth, operated on 23 June 2025	2025-11-01 00:00:00	21
Still stable	2025-11-02 00:00:00	16

Vital status (1=alive,2=dead)	Oxaliplatin dose reduction (0= No, 1=once time, 2= more than once)
1	0
1	0
1	0
1	0
1	2
1	0
1	2
1	0
1	1
1	0
1	0
1	0
1	0
1	1
1	0
1	0
1	1
1	0
1	0
1	0
1	0
1	0
1	0
1	0
1	0
1	1
1	0
1	0
1	0
1	0
1	0
1	0
1	0
1	0
1	0
1	0
1	0
1	0
1	0
1	1
1	2
1	0
1	0
1	0
1	0
1	0
1	0
1	0
1	0
1	1
1	2

1	0
1	0
1	0
1	1
1	0
1	0
1	0
1	1
1	0
1	0
1	1
1	1
1	0
1	0
1	0
1	1
1	0
1	0
1	2
1	2
1	1
1	1
1	0
1	2
1	0
1	0
1	0
1	0
1	0
1	0
1	0
1	2
1	0
1	0
1	0
1	1
1	1
1	0
1	0
1	1
1	0
1	0
1	0
1	0
1	0
1	1
1	0
1	0
1	0
1	0

Oxaliplatin dose (per cycle)	Capecitabine dose reduction (0= No, 1=once time, 2= more than once)
100	0
100	0
100	0
100	0
75	0
100	0
90	1
100	0
90	1
100	0
100	0
100	0
91,7	0
94	0
100	0
100	0
85	0
100	0
100	1
100	0
100	0
100	0
100	0
100	0
100	0
94,4	0
100	0
100	0
100	0
100	0
100	0
100	0
100	0
100	0
100	0
85	0
100	0
100	0
100	0
94,7	1
75	0
100	0
100	0
90	0
100	1
100	0
100	0
100	0
100	0
94	0
83,3	0

100	0
100	0
100	0
94,1	0
100	0
100	1
100	0
94,4	0
100	0
100	0
92,3	0
79	1
100	0
100	0
100	0
78,9	0
100	0
100	0
75	2
85,7	0
80	0
88,9	1
100	0
833	1
91	0
100	0
100	0
100	0
100	0
100	0
100	0
83,3	0
100	0
100	0
100	0
94,7	0
100	0
100	0
100	0
75	0
100	0
100	1
100	0
100	0
100	0
75	0
100	0
100	0
100	0
100	0

Capecitabine dose (per cycle)
100
100
100
100
100
100
83,3
100
83,3
100
100
100
100
100
100
100
100
100
80
100
100
100
100
100
100
100
100
100
100
100
100
100
100
100
100
100
100
100
100
80
100
100
100
100
90
100
100
100
100
100
100

100
100
100
100
100
80
100
100
100
100
100
80
100
100
100
100
100
100
75
100
100
80
100
80
100
100
100
100
100
100
100
100
100
100
100
100
100
100
100
100
100
100
100
100
100
100
100
100
100
100

Hypersensitivity reaction to chemotherapy (0=No, 1= grade 1, 2= grade 2, 3=grade 3)
1
0
0
0
3
0
0
0
1
0
0
0
0
1
0
0
0
0
0
0
0
0
0
0
0
0
1
0
1
0
0
0
1
0
2
0
0
0
0
1
0
0
0
0
1
0
0
0
0
0
0

0
0
2
1
0
0
0
0
0
1
1
0
0
2
0
0
0
0
0
0
0
0
0
0
1
0
0
0
0
0
1
0
0
0
1
0
0
0
0
0
1
0
0
2
0
0
2
0
0
0

Fatigue, grade 1 (1=yes, 0=no)	Fatigue, grade 2(1=yes, 0=no)	Fatigue, grade 3 (1=yes, 0=no)
1	0	0
1	1	0
0	0	0
0	1	0
1	1	0
1	0	0
1	0	0
0	0	0
1	0	0
0	0	0
1	0	0
0	0	0
0	0	0
1	1	0
0	0	0
1	0	0
1	0	0
0	1	0
1	0	0
0	0	0
1	0	0
0	0	0
1	0	0
0	0	0
0	0	0
1	0	0
1	1	0
0	0	0
0	0	0
0	0	0
0	0	0
1	0	0
0	0	0
0	0	0
1	0	0
0	0	0
0	0	0
0	0	0
1	0	0
0	0	0
1	0	0
0	1	0
0	0	0
1	0	0
0	0	0
0	1	0
0	0	0
1	0	0
1	0	0
0	0	0
0	0	0

0	0	0
0	0	0
1	0	0
1	1	0
1	0	0
0	0	0
0	0	0
1	0	0
0	0	0
0	0	0
0	0	0
1	0	0
0	0	0
1	0	0
0	1	0
1	0	0
0	0	0
1	0	0
1	0	1
0	0	0
1	0	0
0	0	0
0	0	0
0	0	0
1	0	0
0	0	0
0	0	0
1	0	0
1	0	0
0	0	0
1	1	0
1	0	0
0	0	0
0	0	0
0	0	0
1	0	0
0	0	0
0	0	0
0	0	0
0	0	0
0	0	0
1	0	0
1	0	0
0	0	0
0	0	0
0	0	0
0	0	0
1	0	0
1	0	0
0	0	0

Peripheral neuropathy (hand paresthesia), grade 1 (1=yes, 0=no)
1
1
1
0
1
1
0
0
1
0
0
0
1
1
1
1
0
0
1
0
1
0
1
0
0
1
1
0
0
0
0
1
0
0
0
0
0
0
1
1
1
0
1
0
1
0
1
0
1
1
1

1
1
1
1
1
1
0
1
1
0
1
0
0
0
1
0
0
0
1
1
1
1
0
0
1
0
0
1
1
1
1
1
1
0
0
0
1
0
0
1
0
1
1
1
0
0
1
1
1
1

Peripheral neuropathy (hand paresthesia), grade 2 (1=yes, 0=no)
0
1
0
0
1
0
0
0
1
0
0
0
1
0
0
0
0
0
1
0
0
0
1
0
0
1
1
0
0
0
0
0
0
0
0
0
0
0
0
0
0
0
0
0
1
0
0
0
0
0
0

0
0
0
0
1
1
0
0
0
0
0
0
0
0
0
0
0
0
0
0
0
0
0
0
0
0
0
0
0
0
0
0
0
0
0
0
0
0
0
0
0
0
0
0
0
0
0
0
0
0

Peripheral neuropathy (hand paresthesia), grade 3 (1=yes, 0=no)
0
0
0
0
0
0
0
0
0
0
0
0
1
0
0
0
0
0
0
0
0
0
0
0
0
0
0
0
0
0
0
0
0
0
0
0
0
0
0
0
0
0
0
0
0
0
0
0
0
0
0

0
0
0
1
1
0
0
0
0
0
0
0
0
0
0
0
0
0
0
0
0
0
0
0
0
0
0
0
0
0
0
0
0
0
0
0
0
0
0
0
0
0
0
0
0
0
0
0
0
0

Peripheral neuropathy (hand paresthesia), grade 4 (1=yes, 0=no)
0
0
0
0
0
0
0
0
0
0
0
0
0
0
0
0
0
0
0
0
0
0
0
0
0
0
0
0
0
0
0
0
0
0
0
0
0
0
0
0
0
0
0
0
0
0
0
0
0
0
0

0
0
0
0
0
0
0
0
0
0
0
0
0
0
0
0
0
0
0
0
0
0
0
0
0
0
0
0
0
0
0
0
0
0
0
0
0
0
0
0
0
0
0
0
0
0
0
0
0
0

Hand–foot syndrome, grade 1 (1=yes, 0=no)	Hand–foot syndrome, grade 2 (1=yes, 0=no)
0	0
1	1
0	0
0	0
1	1
1	0
0	0
0	0
1	1
0	0
0	0
0	0
1	1
0	0
0	0
0	0
0	0
0	0
1	1
0	0
0	0
0	0
1	0
0	0
0	0
1	0
1	0
0	0
0	0
0	0
0	0
1	0
0	0
0	0
0	0
0	0
0	0
0	0
1	0
1	0
1	0
0	0
1	0
0	0
1	0
0	0
1	0
0	0
0	0
0	0
0	0

0	0
1	0
0	0
1	0
1	0
1	1
0	0
1	0
1	0
0	0
1	0
0	0
0	0
0	0
1	0
0	0
0	0
0	0
1	0
0	0
1	0
1	0
0	0
0	0
0	0
0	0
0	0
1	0
1	0
1	0
1	0
1	0
1	0
0	0
0	0
0	0
1	0
0	0
0	0
0	0
0	0
1	0
0	0
1	0
0	0
0	0
0	0
1	0
0	0
0	0

Hand–foot syndrome, grade 3 (1=yes, 0=no)	Hand–foot syndrome, grade 4 (1=yes, 0=no)
0	0
0	0
0	0
0	0
0	0
0	0
0	0
0	0
0	0
0	0
0	0
0	0
1	0
0	0
0	0
0	0
0	0
0	0
0	0
0	0
0	0
0	0
0	0
0	0
0	0
0	0
0	0
0	0
0	0
0	0
0	0
0	0
0	0
0	0
0	0
0	0
0	0
0	0
0	0
0	0
0	0
0	0
0	0
0	0
0	0
0	0
0	0
0	0
0	0
0	0
0	0

0	0
0	0
0	0
1	0
0	0
0	0
0	0
0	0
0	0
0	0
0	0
0	0
0	0
0	0
0	0
0	0
0	0
0	0
0	0
0	0
0	0
0	0
0	0
0	0
0	0
0	0
0	0
0	0
0	0
0	0
0	0
0	0
0	0
0	0
0	0
0	0
0	0
0	0
0	0
0	0
0	0
0	0
0	0
0	0
0	0
0	0
0	0
0	0
0	0
0	0

Leukopenia, grade 1 (1=yes, 0=no)	Leukopenia, grade 2 (1=yes, 0=no)
0	0
0	0
0	0
0	0
0	0
0	0
0	0
0	0
0	0
1	0
0	0
0	0
0	0
1	1
1	0
1	0
0	0
0	0
0	0
0	0
1	0
0	0
0	0
0	0
0	0
0	1
0	1
0	0
1	0
0	0
1	0
1	0
0	0
0	0
1	0
0	0
0	0
0	0
0	0
0	0
0	0
0	0
0	0
0	0
1	0
0	0
0	0
0	0
0	0
0	0
1	0

1	0
1	0
0	0
0	0
0	0
0	0
1	0
0	1
1	0
0	0
0	0
0	0
1	0
0	0
0	0
1	0
0	0
0	0
1	0
0	0
0	0
1	0
0	0
1	0
0	0
1	0
0	0
0	0
0	0
0	0
1	0
0	0
1	0
0	0
1	0
0	0
1	0
0	0
0	0
1	0
0	0
0	0
0	0
0	0
0	0
0	0
0	0
0	0
0	0
0	0

Leukopenia, grade 3 (1=yes, 0=no)	Leukopenia, grade 4 (1=yes, 0=no)
0	0
0	0
0	0
0	0
0	0
0	0
0	0
0	0
1	0
0	0
0	0
0	0
0	0
0	0
0	0
0	0
0	0
0	0
0	0
0	0
0	0
0	0
0	0
0	0
0	0
0	0
0	0
0	0
0	0
0	0
0	0
0	0
0	0
0	0
0	0
0	0
0	0
0	0
0	0
0	0
0	0
0	0
0	0
0	0
0	0
0	0
0	0
0	0
0	0
0	0
0	0

0	0
0	0
0	0
0	0
0	0
0	0
0	0
0	0
0	0
0	0
0	0
0	0
0	0
0	0
0	0
0	0
0	0
0	0
0	0
0	0
0	0
0	0
0	0
0	0
0	0
0	0
0	0
0	0
0	0
0	0
0	0
0	0
0	0
0	0
0	0
0	0
0	0
0	0
0	0
0	0
0	0
0	0
0	0
0	0
0	0
0	0
0	0
0	0
0	0
0	0

Thrombocytopenia, grade 1 (1=yes, 0=no)	Thrombocytopenia, grade 2 (1=yes, 0=no)
0	1
0	0
0	0
0	0
1	0
0	0
1	0
0	0
0	0
0	0
0	0
0	0
1	0
0	1
0	0
0	0
0	0
0	0
0	0
0	0
1	0
0	0
0	0
0	0
0	0
0	0
0	0
0	0
0	0
0	0
1	0
1	0
1	0
0	0
1	0
0	0
0	0
0	0
0	0
0	0
1	0
0	0
0	0
0	0
0	0
0	0
0	0
0	0
0	0
1	1
1	0

0	0
1	0
1	1
1	1
0	0
0	0
0	0
0	0
0	0
0	0
1	0
0	0
0	0
0	0
1	0
1	1
0	0
1	1
1	0
1	1
1	1
1	1
0	0
1	0
0	0
1	0
1	0
0	0
0	0
1	0
0	0
1	0
0	0
0	0
1	0
1	0
0	0
0	0
0	0
1	1
1	0
0	0
0	0
0	0
0	0
1	1
1	0
0	0
1	0
0	0

Thrombocytopenia, grade 3 (1=yes, 0=no)	Thrombocytopenia, grade 4 (1=yes, 0=no)
0	0
0	0
0	0
0	0
1	0
1	0
0	0
0	0
0	0
0	0
0	0
0	0
0	0
0	0
0	0
0	1
0	0
0	0
0	0
0	0
0	0
0	0
1	0
0	0
0	0
0	0
1	0
0	0
0	0
0	0
0	0
0	0
0	0
0	1
0	0
0	0
0	0
0	0
0	0
0	0
0	0
0	0
0	0
0	0
0	0
0	0
0	0
0	0
0	0
0	0
0	0

0	0
0	0
0	0
0	0
0	0
1	0
0	0
0	0
0	0
0	0
0	0
0	0
0	0
0	0
0	0
1	0
0	0
0	0
1	0
0	0
0	0
0	1
0	0
0	0
0	0
0	0
0	0
0	0
0	0
0	0
0	0
1	0
0	0
0	0
0	0
0	0
0	0
0	0
0	0
0	0
1	0
0	0
0	0
0	0
0	0
0	0
0	0
0	0
0	0
0	0

Anemia, grade 1 (1=yes, 0=no)	Anemia, grade 2 (1=yes, 0=no)	Anemia, grade 3 (1=yes, 0=no)
1	0	0
0	0	0
1	0	0
0	0	0
1	0	1
0	0	0
1	1	0
0	0	0
0	0	0
1	0	0
0	0	0
0	0	0
1	0	0
0	0	0
1	1	0
0	0	0
0	0	0
0	0	0
0	0	0
0	0	0
1	1	0
0	0	0
0	0	0
0	0	0
1	1	0
0	0	0
0	0	0
0	0	0
1	0	0
0	0	0
1	0	0
0	0	0
0	0	0
1	0	0
1	0	0
0	0	0
0	0	0
0	0	0
0	0	0
0	1	0
1	0	0
0	0	0
0	0	0
0	0	0
0	0	0
0	0	0
1	0	0
0	0	0
0	0	0
0	0	0
1	0	0

0	0	0
1	0	0
1	0	0
0	0	0
0	0	0
0	0	0
1	1	0
1	1	0
0	0	0
0	0	0
0	0	0
1	0	0
0	0	0
0	0	0
1	0	0
1	0	0
0	0	0
0	0	0
0	1	0
1	0	1
0	0	0
1	0	0
0	0	0
1	0	0
0	0	0
0	0	0
1	0	0
0	0	0
0	0	0
1	1	0
0	0	0
0	0	0
0	0	0
0	0	0
0	0	0
1	0	0
1	0	0
1	0	0
0	0	0
1	0	0
1	0	1
0	0	0
0	0	0
1	0	0
0	0	0
1	0	0
1	0	1
0	0	0
0	0	0
0	0	0

Anemia, grade 4 (1=yes, 0=no)	Elevated liver enzymes, grade 1 (1=yes, 0=no)
0	0
0	1
0	1
0	0
0	0
0	0
0	1
0	0
0	1
0	1
0	1
0	0
0	1
0	0
0	1
0	1
0	0
0	1
0	0
0	0
0	1
0	1
0	1
0	0
0	1
0	1
0	1
0	0
0	0
0	1
0	1
0	0
0	1
0	1
0	1
0	0
0	0
0	0
0	0
0	1
0	1
0	0
0	0
0	0
0	0
0	0
0	1
0	0
0	0
0	1
0	1

0	0
0	1
0	0
0	0
0	0
0	1
0	0
1	0
0	0
0	0
0	0
0	0
0	0
0	0
0	0
0	1
0	0
0	1
0	1
0	1
0	0
0	0
0	1
0	1
0	1
0	1
0	1
0	0
0	0
0	1
0	0
0	0
0	1
0	0
0	0
0	1
0	1
0	0
0	0
0	1
0	0
0	1
0	0
0	0
0	0
0	0
0	0
0	0
0	0
0	0

Elevated liver enzymes, grade 2 (1=yes, 0=no)	Elevated liver enzymes, grade 3 (1=yes, 0=no)
0	0
1	0
0	0
1	0
0	0
0	0
0	0
0	0
0	0
0	0
1	0
0	0
0	0
0	0
0	0
0	0
0	0
0	0
0	0
0	0
1	0
0	0
0	0
0	0
0	0
0	0
1	0
0	0
0	0
0	0
0	0
0	0
0	0
0	0
0	0
0	0
0	0
0	0
0	0
0	0
0	0
0	0
0	0
0	0
0	0
0	0
0	0
0	0
0	0
0	0
0	0

0	0
0	0
0	1
0	0
0	0
0	0
0	0
0	0
0	0
0	0
0	1
0	0
0	0
0	0
0	0
0	0
0	0
0	1
0	0
1	0
0	0
0	0
0	0
0	0
0	0
1	0
0	0
0	0
0	0
0	0
0	0
0	0
0	0
0	0
0	0
0	0
0	0
0	0
0	0
0	0
0	0
0	0
0	0
0	0
0	0
0	0
0	0
0	0
0	0
0	0

Elevated liver enzymes, grade 4 (1=yes, 0=no)	Abdominal pain, grade 1 (1=yes, 0=no)
0	0
0	1
0	1
0	0
0	0
0	0
0	1
0	0
0	0
0	0
0	1
0	0
0	0
0	0
0	1
0	1
0	0
0	1
0	0
0	0
0	1
0	0
0	1
0	0
0	0
0	0
0	1
0	0
0	0
0	1
0	1
0	0
0	1
0	1
0	0
0	0
0	0
0	0
0	0
0	0
0	0
0	0
0	0
0	0
0	0
0	0
0	1
0	0
0	1
0	0
0	0

0	0
0	0
0	0
0	0
0	1
0	0
0	0
0	1
0	1
0	0
0	1
0	0
0	0
0	0
0	0
0	1
0	0
0	1
0	0
0	1
0	0
0	0
0	1
0	1
0	0
0	0
0	1
0	0
0	0
0	1
0	1
0	0
0	1
0	0
0	0
0	1
0	1
0	0
0	0
0	0
0	1
0	0
0	0
0	0
0	0
0	1
0	0
0	0
0	1
0	0

Abdominal pain, grade 2 (1=yes, 0=no)	Abdominal pain, grade 3 (1=yes, 0=no)
0	0
0	0
0	0
0	0
0	0
0	0
1	0
0	0
0	0
1	0
0	0
0	0
0	0
0	0
0	0
0	0
0	0
0	0
0	0
0	0
0	0
0	0
0	0
0	0
0	0
0	0
1	0
0	0
0	0
0	0
0	0
0	0
0	0
0	0
0	0
0	0
0	0
0	0
0	0
0	0
0	0
0	0
0	0
0	0
1	0
0	0
0	0
0	0
0	0
0	0
0	0

0	0
0	0
0	0
0	0
0	0
0	0
0	0
0	0
0	0
0	0
0	1
0	0
0	0
0	0
0	0
0	0
0	0
0	0
0	0
0	0
0	0
0	0
0	0
0	0
1	0
1	0
0	0
0	0
0	0
0	0
0	0
0	0
0	0
0	0
0	0
0	0
0	0
0	0
0	0
0	0
0	0
0	0
0	0
0	0
0	0
0	0
0	0
0	0
0	0
0	0

Abdominal pain, grade 4 (1=yes, 0=no)	Nausea/vomiting, grade 1 (1=yes, 0=no)
0	0
0	1
0	1
0	1
0	0
0	0
0	1
0	0
0	0
0	1
0	0
0	0
0	1
0	0
0	1
0	0
0	0
0	1
0	0
0	0
0	1
0	1
0	0
0	0
0	1
0	0
0	1
0	1
0	0
0	1
0	1
0	0
0	1
0	1
0	1
0	0
0	0
0	0
0	0
0	1
0	0
0	0
0	0
0	1
0	1
0	0
0	1
0	0
0	0
0	0
0	0

0	0
0	1
0	0
0	0
0	0
0	1
0	1
0	0
0	0
0	0
0	1
0	0
0	0
0	0
0	0
0	0
0	0
0	0
0	0
1	0
0	0
0	0
0	1
0	1
0	1
0	0
0	1
0	0
0	0
0	1
0	0
0	0
0	0
0	1
0	0
0	0
0	1
0	0
0	0
0	0
0	0
0	0
0	0
0	0
0	0
0	0
0	0
0	0
0	0
0	1

Nausea/vomiting, grade 2 (1=yes, 0=no)	Nausea/vomiting, grade 3 (1=yes, 0=no)
0	0
1	0
1	0
0	0
0	0
0	0
0	1
0	0
0	0
1	0
0	0
0	0
0	0
0	0
0	0
0	0
0	0
0	0
0	0
0	0
1	0
1	0
0	0
0	0
1	0
0	0
0	1
0	0
0	0
0	0
0	0
0	0
0	0
0	0
0	0
0	0
0	0
0	0
0	0
0	0
0	1
0	0
0	0
0	0
0	0
0	0
0	0
1	0
0	0
1	0
1	0

0	0
1	0
0	0
0	0
0	0
0	0
0	0
0	0
0	0
0	0
0	0
0	0
0	0
0	0
0	0
1	0
0	0
0	0
0	0
0	1
0	0
1	0
0	0
0	0
0	0
0	0
0	0
0	0
0	0
0	0
0	0
0	0
0	0
0	0
0	0
0	0
0	0
0	0
1	0
0	0
0	0
0	0
0	0
0	1
1	0
0	0
0	0
0	0
0	0
0	0

Nausea/vomiting, grade 4 (1=yes, 0=no)	Diarrhea, grade 1 (1=yes, 0=no)	Diarrhea, grade 2 (1=yes, 0=no)
0	0	0
1	0	0
0	0	0
0	0	0
0	0	0
0	1	0
0	0	0
0	0	0
0	0	0
0	0	0
0	0	0
0	0	0
0	0	0
0	0	0
0	1	0
0	0	0
0	0	0
0	0	0
0	0	0
0	0	0
0	0	0
0	0	0
0	0	0
0	0	0
0	0	0
0	0	0
0	0	0
0	0	0
0	0	0
0	0	0
0	0	0
0	0	0
0	0	0
0	1	0
0	0	0
0	0	0
0	1	0
0	0	0
0	0	0
0	0	0
0	0	0
0	0	0
0	0	0
0	0	0
0	1	0
0	1	0
0	0	0
0	0	0
0	0	0
0	1	0
0	0	0

0	0	0
0	1	1
0	0	0
0	0	0
0	0	0
0	0	0
0	0	0
0	0	0
0	1	1
0	1	0
0	1	0
0	0	0
0	0	0
0	0	0
0	0	0
0	1	0
0	0	0
0	0	0
0	1	1
0	1	0
0	0	0
0	0	0
0	1	0
0	0	0
0	1	0
0	0	0
0	0	0
0	1	1
0	0	1
0	0	0
0	1	0
0	1	0
0	0	0
0	0	0
0	0	0
0	0	0
0	0	1
0	0	0
0	1	0
0	1	0
0	1	0
0	0	0
0	0	0
0	1	0
0	0	0
0	0	0
0	1	0
0	1	0
0	0	0
0	0	0

Diarrhea, grade 3 (1=yes, 0=no)	Diarrhea, grade 4 (1=yes, 0=no)	Anal pain (any grade) (1=yes, 0=no)
0	0	0
0	0	0
0	0	0
0	0	0
0	0	1
0	0	0
0	0	0
0	0	0
0	0	0
0	0	0
0	0	0
0	0	0
0	0	0
0	0	0
0	0	0
0	0	0
0	0	0
0	0	0
0	0	0
0	0	0
0	0	0
0	0	0
0	0	0
0	0	0
0	0	0
0	0	0
0	0	0
0	0	0
0	0	0
0	0	0
0	0	0
0	0	0
0	0	0
0	0	0
0	0	0
0	0	0
0	0	0
0	0	0
0	0	0
0	0	0
0	0	0
0	0	0
0	0	0
0	0	0
0	0	0
0	0	0
0	0	0
0	0	0
0	0	0
0	0	0
1	0	0

0	0	0
0	0	0
0	1	0
0	0	0
0	0	0
0	0	0
0	0	0
0	0	3
0	0	0
0	0	0
0	0	0
0	0	0
0	0	0
0	0	0
0	0	0
0	0	0
0	0	1
0	0	0
0	0	0
0	0	0
0	0	0
0	0	0
0	0	0
0	0	0
0	0	0
0	0	0
0	0	0
0	0	0
0	0	0
1	0	0
0	0	0
0	0	0
0	0	0
0	0	0
0	0	0
0	0	0
0	0	0
0	0	0
0	0	0
0	0	0
0	0	0
0	0	0
0	0	0
0	0	1
0	0	0
0	0	0
0	0	0
0	0	0
0	0	0
0	0	1
